# New Antibacterial Paper Made of Silver Phosphate Cellulose Fibers: A Preliminary Study on the Elimination of *Staphylococcus aureus* Involved in Diabetic Foot Ulceration

**DOI:** 10.1155/2020/1304016

**Published:** 2020-01-08

**Authors:** Virginie Blanchette, Dan Belosinschi, Thanh Tung Lai, Lyne Cloutier, Simon Barnabé

**Affiliations:** ^1^Université du Québec à Trois-Rivières, Podiatric Medicine Program, 3351, Boul. des Forges, C.P.500, Trois-Rivières, Québec G8Z 4M3, Canada; ^2^Innofibre, Cégep de Trois-Rivières, 3351 Boul. des Forges, Trois-Rivières, Québec G9A 5E6, Canada; ^3^Université du Québec à Trois-Rivières, Lignocellulosic Material Research Center, 3351, Boul. des Forges, C.P.500, Trois-Rivières, Québec G8Z 4M3, Canada; ^4^Université du Québec à Trois-Rivières, Nursing Department, 3351, Boul. des Forges, C.P.500, Trois-Rivières, Québec G8Z 4M3, Canada; ^5^Université du Québec à Trois-Rivières, Department of Biochemistry, Chemistry and Physics, 3351, Boul. des Forges, C.P.500, Trois-Rivières, Québec G8Z 4M3, Canada

## Abstract

**Aim:**

To evaluate in vitro the antibacterial effect of a paper made of silver phosphate cellulose fibers (SPCF) on *Staphylococcus aureus*, the most common diabetic foot ulceration (DFU) pathogen when compared with other common commercial products.

**Methods:**

The antibacterial activity of SPCF samples was evaluated through time with cell counting on agar plates. SPCF samples were then compared with commercial wound care products currently in use in DFU treatments (Silvercel™, Acticoat 7, and Aquacel Ag Extra^TM^) through time on agar plates (growth inhibition zones).

**Results:**

After 6 hours, there was no viable bacterial cell detected on either plate (*p* < 0.05). There was a net growth inhibition zone for SPCF samples but no significant difference between the two silver concentrations. Compared with common commercial products, SPCF paper provides results equal to Acticoat 7 (*p* < 0.05) and superior to Aquacel AG Extra^TM^ and Silvercel™ at lower silver concentrations (*p* < 0.001).

**Conclusions:**

These results have shown the efficiency of SPCF paper to eliminate *Staphylococcus aureus* in these conditions. SPCF papers are effective when compared with other common commercial products and could have an industrial potential in wound care. Infected DFU could benefit from the antibacterial effectiveness of SPCF, but more relevant experimentations related to foot ulcers are needed.

## 1. Introduction

Diabetes foot infection (DFI) is a widespread, complex, and costly problem to treat. It is one of the major complications of diabetic foot ulcers (DFU), which can lead to lower extremity amputation and mortality [1–3]. The 5-year mortality rate following a DFU episode is between 43% and 55% and up to 74% with a lower-extremity amputation [4]. DFI is considered to be the predominant cause of hospitalization in patients with diabetes, accounting for about 20% of total diabetes-related hospital admissions in North America [2]. The new wound infection continuum and DFI studies have shown that DFU must be automatically considered infected and that healthcare providers should apply the latest recommendations for biofilm-based wound care and holistic infection management [5–8]. DFI is caused by multiple bacterial strains, but the main pathogen involved in DFU is *Staphylococcus aureus* [9, 10]. However, with the different stages of the continuum and healing delays caused by dense colonization, bacterial tolerance to several forms of treatments, and its subsequent immune response, several gaps remain in understanding the management of this infection, particularly in DFI caused by biofilms. There is a distinct clinical need to understand DFI caused by biofilm, and the scientific community needs more evidence concerning its role and impacts in order to manage it optimally [5, 11, 12]. Therefore, this study evaluate the effectiveness of another material, made with old molecule, against biofilm-forming bacterial strains and safe for the patient [12–15]. Several elements can interfere with research on wound care products, such as unknown antibacterial molecules effectiveness, standardized methods, and regulatory guidelines to establish the performance of targeted molecules. These few examples limit the implication of the industry and end up driving up the cost of research and development, despite the need for clinical trials [16]. In that context, the objective of this study was to conduct a preliminary examination of a potential antibacterial material that uses pulp and paper expertise for biomedical purposes. Cellulose is a well-known, low-cost raw material widely used in wound care, while silver is also recognized for its antibacterial effectiveness [17–20]. The wound care community remains skeptical and conservative about the widespread use of antibacterial dressings and silver, even with the new evidence proposed in the wound infection continuum [5, 10]. This can be explained by bacterial resistance due to overuse or inappropriate use and cytotoxicity of antibacterial molecules, which are barriers to healing and harmful for the patient [21]. These products are also expensive [5, 21–23]. Few occurrences of resistance to silver have been reported in the medical literature, and the cellular sensitivity is correlated with the level of silver release and exposure [14, 15, 24, 25]. The material we tested, a paper composed of silver phosphate cellulose fibers (SPCF), is composed of silver ions, which is the form of silver already effective against bacteria and fungi through enzymatic systems, blocking respiratory cycle and destroying cell wall membrane [26, 27].

In the context where DFUs should always be considered infected, a recent Cochrane intervention review suggests that DFU healing will be superior with antibacterial wound dressing than without it [17]. These results correspond with 119 additional healing events with an antibacterial dressing in 1000 participants (95% CI from 51 to 191 more) [17]. Thereby, the aim of this study is to evaluate the antibacterial effect of silver phosphate cellulose fiber (SPCF) paper on planktonic *Staphylococcus aureus* potentially film-forming, by standard susceptibility testing in vitro. The objectives are to (1) determine the antibacterial effect of two concentrations of SPCF by time, from a stock solution of *Staphylococcus aureus* and (2) compare SPCF samples to commercial products generally used in wound care for DFU (Silvercel™, Acticoat 7, and Aquacel Ag Extra™), which also use silver as an antibacterial agent, on an agar plate by growth inhibition zone (clear zone).

## 2. Materials and Methods

### 2.1. Media and Bacterial Strain

The media used in this study were tryptic-soy agar (TSA) and tryptic-soy broth (TSB) (Difco Laboratories, Detroit, MI, USA). Media were autoclaved for 30 min at 120°C*. Staphylococcus aureus* (ATCC® 25923™), provided by the microbiology laboratory of the Université du Québec à Trois-Rivières was the strain specifically selected for its biofilm forming potential, considering its adhesion properties [28]. The strain was grown on TSA at 37°C.

### 2.2. Preparation of SPCF Samples

**Figure 1:**
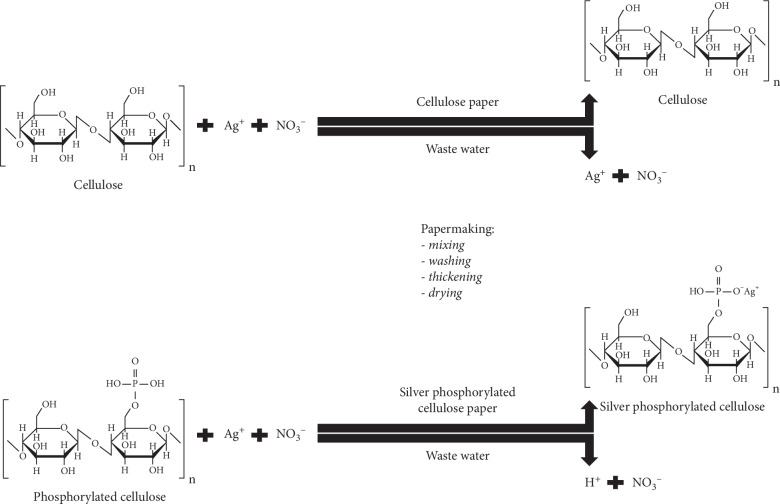
SPCF formation: interactions of silver ions with cellulose fibers.

**Figure 2:**
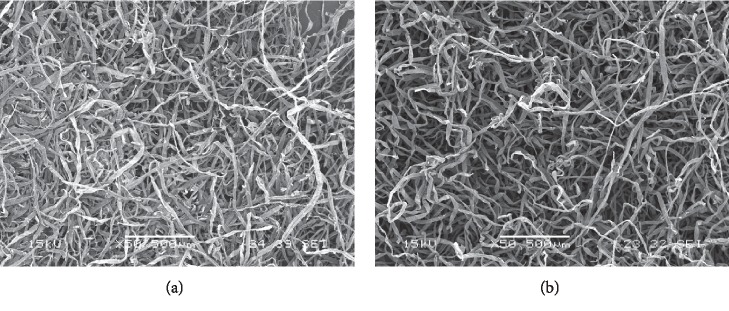
(a) Unmodified cellulose fibers and (b) SPCF (scanning electron microscopy (SEM), JSM T300, JEOL).

**Figure 3:**
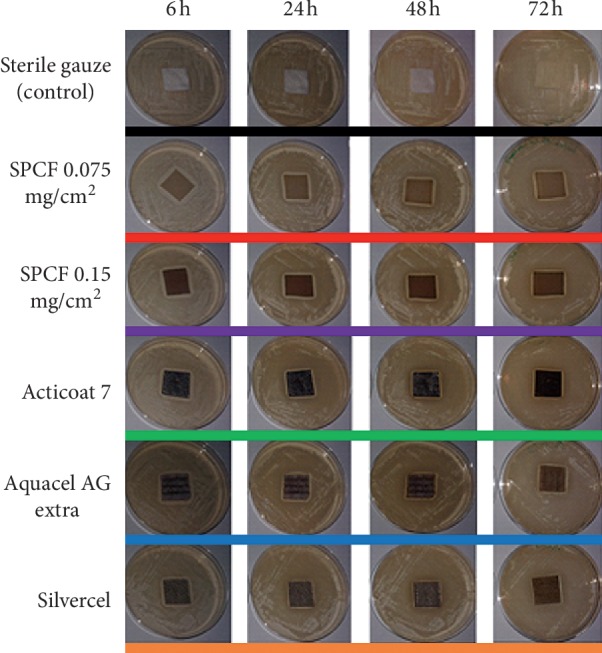
Growth inhibition representations for control, SPCF samples, and commercial products on *Staphylococcus aureus* at 6, 24, 48, and 72 h.

Bleached softwood Kraft fibers (provided by Kruger Trois-Rivières Mill, Trois-Rivières, Canada) were phosphorylated using an original method developed by one of the authors, Dan Belosinschi [29]. The phosphate moiety grafted at the fibers' surface electronegativity charges in water and behaves as an adsorption site for cations like silver ions. Therefore, an isotherm of adsorption was conducted at room temperature for 15 minutes by contacting the phosphorylated fibers with silver nitrate, AgNO3 0.1 N (Sigma-Aldrich, St. Louis, USA), in deionized water. An excess of 20% (weight/weight) of silver was dosed to reach the target values of 0.075 and 0.15 mg/cm^2^ (mg of silver to cm2 of paper). For this preliminary testing, we chose the amount of silver for SPCF's samples based on the commercial dressings, chosen as comparisons for the second objective of this experiment (Table 1). According to a previous publication, theoretical exact amount of ionic silver can be calculated from total phosphorus charges (our SPCF sample was 5000 mmoles/kg); practical adjustment was done to obtain targeted concentrations [33]. The exact amount of ionic silver was determined by analysis with spectrometry (plasma microwaves atomic emission spectrometer, MP-AES) [33]. At the end of adsorption isotherm, the fibers were thoroughly washed with deionized water to remove the nitrate and the free silver ions (Figure 1). Finally, hand sheet papers with a basis weight of 60 g/m^2^ were made from silver phosphate cellulose fibers (SPCF) using the standard method of the pulp and paper industry—Tappi T 205 sp-02. SPCF keeps their morphology from unmodified cellulose fibers, but mechanical and surface properties are different (Figure 2). For example, SPCF strength properties are very low, but that can be compensated by mixing SPCF and unmodified cellulose fibers to keep its original properties with antibacterial properties [29, 34, 35]. SPCF samples had a brown color (see samples represented in Figure 3). It is well known that substrate treated with AgNO_3_ exhibits brown color in aqueous solution due to excitation of surface plasmon vibrations of silver particles [36]. Samples of 2 × 2 cm from the commercial dressing and from SPCF samples were prepared under sterile conditions in triplicate.

### 2.3. Experimental Protocol

To reach the first objective of this study, a stock solution was prepared with a full loop of *Staphylococcus aureus* transferred to a shake flask of 250 ml with sterile TSB. The incubation was carried out overnight (approximately 18 h) under conditions of 125 rpm agitation and 37°C. Bacterial cell concentration was measured by colony-forming units (CFU)/ml using the plate count agar technique combined with optical density on a spectrophotometer at 600 nm length wave (Cary 5000 UV/Vis Spectrometer, Agilent Technologies, USA). The working culture solution was prepared by diluting the stock in a sterile saline solution (0.85% sodium chloride). An aliquot of 10 ml of working culture solution was distributed to 50 ml Falcon tubes, and SPCF samples were placed on the top of the solution in the tube. Thus, the samples were totally submerged by capillarity and in contact with the liquid surface of the working culture solution without causing the dissolution of cellulosic fibers in the aqueous environment. Sterile gauze (unmodified cellulose fibers) was used as a negative control, and the experiment was performed in triplicate. Sampling was carried out at 0, 3, 6, 9, 12, 24, 36, and 48 h and kept at 4°C for analysis. The final bacterial cell concentration in the sample was measured by the plate count agar technique in triplicate.

For the second objective, the working culture was prepared as described in the first objective, and 10^6^ CFU/ml bacterial cells were inoculated on the surface of TSA in order to obtain a bacterial lawn of *Staphylococcus aureus*. Wound infection is described in the literature as a bacterial load of ≥10^5^ CFUs, and it has been hypothesized that this bacterial load may correspond to infected wounds with biofilm [5, 37]. Then, we hypothesized that the bacterial value for antibacterial testing should be 10^6^ CFUs. SPCF samples should be effective at this bacterial concentration and at least demonstrate an inhibition growth zone. Thereafter, SPCF samples, Silvercel™, Acticoat7, Aquacel Ag Extra™, and gauze (as the negative control) were sterilely placed on the center of the TSA plates and incubated at 37°C. The observations were carried out at 6, 24, 48, and 72 h, and the experiment was performed in triplicate. Growth inhibition zones in the TSA were measured with a digital caliper (Insize Series 1102, Global industrial, USA).

### 2.4. Statistical Methods

For the first objective, statistical variation of results was expressed by means of bacterial cell counts (in log10), with standard deviations of 5% between triplicates, for each sample. For the second objective, the measurement accuracy of the digital caliper was ±0.02 mm with a resolution of 0.01 mm. The variation between sample sizes was measured with a coefficient of correlation (*R* = 0.915), meaning that sample size did not influence the measures. A two-dimensional analysis of variance with repeated measurement (Anova A × Br) and a *t* test mean comparison according to the Dunn–Sidàk correction (which allow multiple comparisons) was performed for some samples to determine any statistically significant differences between the most efficient samples at *p* < 0.05 (Excel, Microsoft Office 2013, USA).

## 3. Results and Discussion

### 3.1. Antibacterial Effect of SPCF

**Figure 4:**
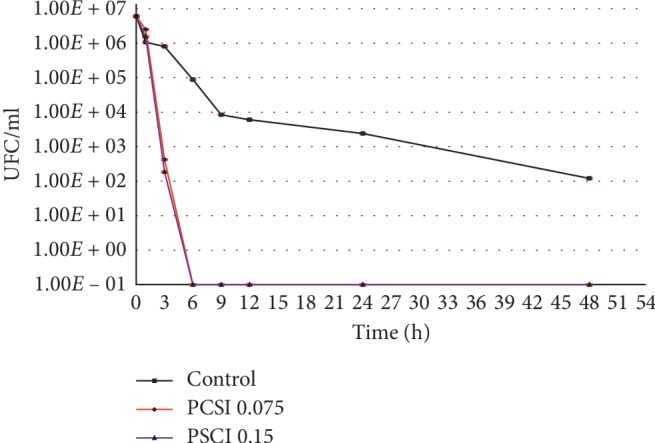
Plot of *Staphylococcus aureus* (CFU/ml for control) and SPCF samples by time.

Figure 4 illustrates the antibacterial effect of two concentrations of SPCF by time, from a stock solution of *Staphylococcus aureus,* in these in vitro conditions. The total viable counts of *Staphylococcus aureus* reveal no survivor cell after 6 hours for both samples. There is a reduction of 1 log_10_ after 1 h, 5 log_10_ after 3 h, and 7 log_10_ after 6 h for both samples. Low standard deviation (between triplicates for each period) indicates a good reproducibility, and that there is no significant difference on the bacterial cell count between samples. There is a significant difference with the control at 3, 9, 12, 24, and 48 h (*p* < 0.05).

### 3.2. Comparisons of SPCF Samples with Commercial Products

**Figure 5:**
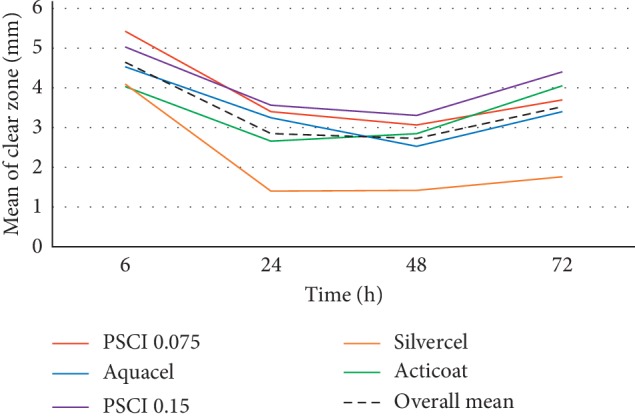
Means of growth inhibition of *Staphylococcus aureus* and overall mean, by time for SPCF samples and commercial products (control is not presented in the graph because it is equal to 0 mm of inhibition).

Figure 4 represents growth inhibition for each sample, by time (6, 24, 48, and 72 h). It was found that all samples prevented *Staphylococcus aureus* growth except for the control sample.  Figure 5 represents the growth inhibition zone for each sample by time, taking into consideration the variations of paper size with standard deviation and the trend with the overall sample means. First, at 6 h, SPCF samples were superior to the overall mean and to commercial products, and Aquacel AG Extra™ was the most efficient commercial sample. Then at 24 h, SPCF samples remained superior and Aquacel AG Extra™ became less effective than Acticoat 7. At 48 h, SPCF samples were still superior and Acticoat 7 was still the most efficient commercial product. Finally, at 72h, SPCF 0.15 mg/cm^2^ was superior and Acticoat 7 remained the most efficient commercial product. Silvercel™ was the least efficient sample for the timing period. In addition, all SPCF samples were superior to the overall mean. Results were significant between samples (*p* < 0.001), for different time measurements (*p* < 0.001) and for both (*p* < 0.01). The antibacterial effect of SPCF samples duration was of a minimum of 72 h according to this second part of the experiment.

Following this trend, samples comparisons were performed between the SPCF samples and the Aquacel AG Extra™ and Acticoat 7. The results presented superior effects of SPCF 0.075 mg/cm^2^ (*p* < 0.001) and 0.15 mg/cm^2^ (*p* < 0.01) when compared with Aquacel AG Extra™, and SPCF 0.15 mg/cm^2^ (*p* < 0.05), when compared with Acticoat 7. There is no significant difference between the SPCF 0.0.075 mg/cm^2^ sample and Acticoat 7. Furthermore, there is no significant difference between SPCF sample concentrations' means of efficiency over time.

## 4. Discussion

DFU is invariably associated with poor healing and susceptibility to recurrent infections, as it is related to the wound infection continuum and biofilm-induced chronic conditions. There is a clinical need to properly manage DFI in order to minimize the impact of bacterial colonization and promote healing. The goals of treating a DFI are the eradication of the infection and the avoidance of soft-tissue loss and amputation. It has been shown that the use of standard antibiotics and antiseptics is not necessarily the optimal treatment option because biofilm is remarkably resistant to these antibacterial agents [3, 11]. The biofilm-based wound care may be a solution [5, 12]. However, this approach requires the use of antibacterial dressing at each debridement, an important step of this approach, which could lead to other problems like bacterial resistance to silver, cellular cytotoxicity, and allergic reactions (hypersensitivity exposure) [14, 20, 38, 39]. Human cytotoxicity to silver is increased with silver concentration and bioaccumulation, causing severe local inflammation and a large intracellular and intercellular edema to fibroblasts and keratinocytes [21, 38]. Silver-containing dressings release silver into the wound, maintaining the elevated concentration (up to 70 ppm) for its antibacterial effectiveness during several days, which is also above the toxic threshold for these important healing cells [21, 40, 41]. These phenomena have been documented with silver exposition and overuse [14, 25, 42, 43]. Therefore, the use of silver needs to be carefully considered. However, the effectiveness of silver is well documented as an effective antibacterial agent against biofilm; it is also able to inhibit 115 clinical bacterial strains isolated from wounds [44–46]. It is the most widely metal used in wound dressing research with cellulose and composite [47]. It induces less bacterial resistance in comparison to antibiotics because of its mechanism of action (multiple sites) [15, 39, 48, 49]. Silver tolerance has been documented in biofilm strains compared with planktonic strains. Studies have documented that higher levels of silver (up to 4 times than minimal inhibitory concentration (MIC)) are needed to treat bacteria that colonize biofilm [50, 51]. Thus, it is imperative to identify alternative products against biofilm with less deleterious effects and more effective ways to deliver ionic components [48].

The evaluation of a new antibacterial paper, silver phosphorylated cellulose fibers, as an innovative way to deliver silver ions rapidly and efficiently in in vitro conditions on planktonic *Staphylococcus aureus* was our goal. Compared with commercial products which sustained release of silver ions at the wound surface from ionic reactions to solubilized AgNO_3_ by wound exudate or moisture of the skin (fluid) and oxygen, SPCF, by its innovative way to incorporating ionic silver, released bactericidal components immediately at the contact area [27, 35]. This presentation maximizes rapid response for bacteria killing while minimizing survival rates and the emergence of mutation and resistance [40]. This is interesting in the context of wound cares, particularly with limitations of dry wounds (less exudative) or diabetic wounds which had tissue hypoxia and limited moisture [52]. The antibacterial effect of silver is related to the amount of silver and the rate of its release, the distribution of silver, and the chemical and physical forms. The affinity of dressing to moisture also influences the bactericidal effect [27, 53]. In the SPCF, the specific amount of silver ions can be calculated precisely (number of phosphorylated groups), which is interesting for maximizing antibacterial activity at lower silver concentration [29, 35].

Similar results between both concentrations (0.075 mg/cm^2^ and 0.15 mg/cm^2^) on *Staphylococcus aureus* are suggesting sufficient concentration of ionic silver during 72h but it can be adjusted at a lower level. The preliminary results of this study show SPCF sample efficiency on *Staphylococcus aureus* lawn within 6 hours, in these in vitro conditions. It demonstrates superior or equal antibacterial effect when compared with commercial products, which can contain as much as the double of silver concentration than the 0.075 mg/cm^2^ SPCF samples. The most effective compared product was Acticoat 7, which contains 1.48 mg/cm^2^, about 10 times more than our highest concentration sample of 0.15 mg/cm^2^.

The large, immediate concentration of silver ions released will become chemically consumed and rapidly inactivated through the formation of chemical complexes with chloride upon contact with organic biological fluids from the wounds [54]. However, in the wound infection continuum, the synergetic effect of several steps over time allows for optimal biofilm management. The mechanical debridement is the first step to manage bioburden (in the wound as well as the biofilm), and the use of antibacterial wound dressing, the final step, is used to prevent biofilm reformation with the remaining film-forming bacteria [5, 45]. In the biofilm life cycle, this represents a few minutes to 4 hours (irreversible biofilm attachment and bacterial adhesion) [55, 56]. The active silver ion must be available as soon as possible to prevent biofilm formation and be bactericidal, as is our new paper, SPCF. Currently, the silver inactivation is compensated for by frequent wound dressing replacements, but this is a costly and time-consuming problem for healthcare professionals and patients, and there is an excess of silver in contact with the wound. There is also a vicious circle to consider: silver ions are responsible for antibacterial activity but the complex of chloride ions produced and then deactivates the silver ions [54]. Nanocrystalline silver, such as in Acticoat 7, has been developed in wound care to allow a slow, stable release of silver ions to fight this phenomenon [57, 58]. However, in this experimental condition, our preliminary results demonstrate that SPCF is more effective than Acticoat 7 (at 6 h), while having a similar overall effect. In this context, our results are promising in regard to the development of a new antibacterial paper intended for human healthcare when rapid antibacterial efficacy is urgently needed, at low cost.

Phosphorylated cellulose fibers can incorporate other metal ions such as zinc (Zn^2+^), which has interesting antibacterial properties against *Staphylococcus aureus* and *Escherichia Coli*, also commonly involved in DFI [59]. Zinc has interesting properties, such as promoting cell migration and proliferation and stimulating epithelialization in wounds [60, 61]. These preliminary results demonstrate the potential of this fiber and may be a new opportunity for the biomedical industry. Phosphorylated cellulose maintains natural touch of unmodified cellulose fibers and can be at all dimensional spectrums of cellulose (macroscopic to nanofibers) [62]. It is a natural polymer, well known for its biocompatibility and biodegradable properties, and can mimic the extracellular matrix. Cellulosic derivatives enhance healing by stimulating several growth factors for fibroblasts and keratinocytes [63, 64]. Cellulose fibers are a cheap and widely available material of choice for sustainable development.

Nevertheless, this study presents several limitations. First, the in vitro conditions of the study and planktonic bacteria testing do not accurately represent the clinical context of a wound. It has been shown that basing a therapy on the conventional susceptibility test (MIC of a bacterium) against biofilm-forming bacteria fails to eradicate the infection. There is still a need for evidence to understand the lack of correlation between MIC results and the therapeutic success of chronic biofilm infections [5, 65]. However, antibacterial testing of our SPCF on biofilm models is needed [65, 66]. Though in some human biofilm cases, studied antimicrobial susceptibility testing on biofilm (minimal biofilm eradication concentration (MBEC)) assays were not superior to MIC to guide antibiotic therapy [67]. In one small study, MBEC of *Staphylococcus aureus* isolated from DFU demonstrated resistance to antibiotics at concentrations 10 to 1000 times higher than those required at MIC, meaning that the antibiotic susceptibility profile cannot be applied to biofilm-established infections, particularly in the case of DFU. In this condition, broad-spectrum antibacterial agents are needed [12, 68, 69]. Finally, few variables were tested in the innovation of the new fibers, which means that further studies are needed to understand the silver ion release, cytotoxicity, and cell interactions in a wound model in vitro and animal model in vivo, as well as time to efficiency and duration, mechanical and physical properties of the SPCF (as a layer), and in combination with other ions such as zinc. This will allow for a better understanding of its potential for DFI treatment. In this perspective, this experiment was conducted to explore new materials with a focus on the DFU biofilm problem, but its potential may be much more extensive. Evidences about this new material are scarce and preliminary, but a strength of this study was the integrated analysis on a health issue combining fundamental and clinical sciences. More relevant experiments related to foot ulcers and this material are needed.

## 5. Conclusions

This is the first in vitro study of silver phosphate cellulose fibers used on *Staphylococcus aureus*; it has demonstrated antibacterial efficiency and effects comparable to commercial wound care products. Therefore, it is the first step exploring a new material for wound cares or in other areas. Further studies are needed to focus on formulation (concentration, combination of molecules for synergic effects, etc.) and on mechanical and physical properties of the new material.

## Figures and Tables

**Table 1:** Characteristics of dressing from the industry with silver and its antimicrobial effect.

Dressing	Material	Silver composition	Antimicrobial effect
Aquacel Ag extra (ConvaTec, Deeside, UK) [30]	2 nonwoven layers of sodium CMC fibers	Approximately 1.2% w/w or 0.17 mg/cm^2^	After 30 min up to 14 days
Acticoat 7 (Smith & Nephew, London, UK) [31]	3 layers of a metallic (nano) crystalline silver-encrusted HDPE mesh alternating with two layers of a rayon polyester nonwoven fabric, bonded at intervals by ultrasonic welding of the HDPE	Approximately 8.4% w/w or 1.48 mg/cm^2^	After 30 min up to 7 days
Silvercel (Acelity, San Antonio, USA) [32]	Nonwoven fabric comprised of a blend of metallic silver-coated nylon fibers and calcium alginate/CMC fibers between two apertured sheets of EMA	Approximately 4% w/w or 1.11 mg/cm^2^ silver	Up to 7 days
Gauze (Alliance, MedicalMart, Mississauga Canada)	2 layers of nonwoven cotton (cellulose)	None	None (control)

CMC: carboxymethylcellulose; w/w: weight/weight; HDPE: high-density polyethylene; EMA: ethylene methyl acrylate.

## Data Availability

The data used to support the findings of this study are available from the corresponding author upon request.

## Supplementary Materials

Supplementary Materials The supplement file is the international patent of phosphorylated lignocellulosic fiber uses and processes of preparation thereof. It provides more fundamental information about the product discussed in the manuscript.
