# Supplementary material for: New Antibacterial Paper Made of Silver Phosphate Cellulose Fibers: A Preliminary Study on the Elimination of Staphylococcus aureus Involved in Diabetic Foot Ulceration
Source: Biomed Res Int. 2020 Jan 8;2020:1304016. doi: 10.1155/2020/1304016 (PMC6973200; doi:10.1155/2020/1304016)
Supplement: Supplementary Materials — The supplement file is the international patent of phosphorylated lignocellulosic fiber uses and processes of preparation thereof. It provides more fundamental information about the product discussed in the manuscript. [file 1304016.f1.pdf]

(12) INTERNATIONAL APPLICATION PUBLISHED UNDER THE PATENT COOPERATION TREATY (PCT)

(19) World Intellectual Property Organization
International Bureau

(43) International Publication Date
21 December 2017 (21.12.2017)

WIPO | PCT

(10) International Publication Number
WO 2017/214719 A1

(51) International Patent Classification:
C08H 8/00 (2010.01) *B01J 20/22* (2006.01)
A61L 15/28 (2006.01) *C02F 1/28* (2006.01)

(21) International Application Number:
PCT/CA2017/050717

(22) International Filing Date:
12 June 2017 (12.06.2017)

(25) Filing Language: English

(26) Publication Language: English

(30) Priority Data:
62/349,207 13 June 2016 (13.06.2016) US

(71) Applicant: **3R VALO, S.E.C.** [CA/CA]; Bureau 503, 355 rue Peel, Montréal, Québec H3C 2G9 (CA).

(72) Inventors: **BELOSINSCHI, Dan**; 360 rue des Seigneurs, Saint-Étienne des Grès, Québec G0X 2P0 (CA). **BROUILLETTE, François**; 6230 de la Montagne, Trois-Rivières, Québec G8Y 5K3 (CA). **SHI, Ying**; c/o Université du Québec à Trois-Rivières, 3351 boul. des Forges, C.P. 500, Trois-Rivières,, Québec G9H 5H7 (CA). **PARADIS, Jean**; 1146 rue Brière, Trois-Rivières, Québec G8V 1N8 (CA). **DOUCET, Josée**; 280 rue Lionel Rheault, Trois-Rivières, Québec G8T 4Y1 (CA).

(74) Agent: **BERESKIN & PARR LLP/S.E.N.C.R.L., S.R.L.**; 40th Floor, 40 King Street West, Toronto, Ontario M5H 3Y2 (CA).

(81) Designated States *(unless otherwise indicated, for every kind of national protection available)*: AE, AG, AL, AM, AO, AT, AU, AZ, BA, BB, BG, BH, BN, BR, BW, BY, BZ, CA, CH, CL, CN, CO, CR, CU, CZ, DE, DJ, DK, DM, DO, DZ, EC, EE, EG, ES, FI, GB, GD, GE, GH, GM, GT, HN, HR, HU, ID, IL, IN, IR, IS, JO, JP, KE, KG, KH, KN, KP, KR, KW, KZ, LA, LC, LK, LR, LS, LU, LY, MA, MD, ME, MG, MK, MN, MW, MX, MY, MZ, NA, NG, NI, NO, NZ, OM, PA, PE, PG, PH, PL, PT, QA, RO, RS, RU, RW, SA, SC, SD, SE, SG, SK, SL, SM, ST, SV, SY, TH, TJ, TM, TN, TR, TT, TZ, UA, UG, US, UZ, VC, VN, ZA, ZM, ZW.

(84) Designated States *(unless otherwise indicated, for every kind of regional protection available)*: ARIPO (BW, GH, GM, KE, LR, LS, MW, MZ, NA, RW, SD, SL, ST, SZ, TZ, UG, ZM, ZW), Eurasian (AM, AZ, BY, KG, KZ, RU, TJ, TM), European (AL, AT, BE, BG, CH, CY, CZ, DE, DK, EE, ES, FI, FR, GB, GR, HR, HU, IE, IS, IT, LT, LU, LV, MC, MK, MT, NL, NO, PL, PT, RO, RS, SE, SI, SK, SM, TR), OAPI (BF, BJ, CF, CG, CI, CM, GA, GN, GQ, GW, KM, ML, MR, NE, SN, TD, TG).

Published:
— *with international search report (Art. 21(3))*

WO 2017/214719 A1

(54) **Title:** PHOSPHORYLATED LIGNOCELLULOSIC FIBERS, USES AND PROCESSES OF PREPARATION THEREOF

(57) **Abstract:** The present disclosure relates to lignocellulosic materials comprising phosphorylated lignocellulosic fibers having an ionic charge in water of about 4000 to about 7000 mmoles/kg, and processes for the preparation thereof. The process comprises reacting lignocellulosic fibers of a lignocellulosic material with a phosphate ester in the presence of urea. The present disclosure further relates to compositions comprising a phosphate ester and at least one of a defoamer and a viscosity reducer or at least one of a C1-C12 alcohol and an ester of a carboxylic acid.

PHOSPHORYLATED LIGNOCELLULOSIC FIBERS, USES AND PROCESSES OF PREPARATION THEREOF

CROSS REFERENCE TO RELATED APPLICATIONS

[001] The present application claims priority from U.S. provisional application no. 62/349,207, filed June 13, 2016, the disclosure of which is hereby incorporated by reference in its entirety.

FIELD OF THE DISCLOSURE

[002] The present disclosure relates to lignocellulosic materials and more particularly to lignocellulosic materials comprising phosphorylated lignocellulosic fibers, uses and processes for the preparation thereof.

BACKGROUND OF THE DISCLOSURE

[003] Phosphorylation of cellulosic substrate using phosphoric acid or its derivative as a phosphorylation agent is known. A main drawback however of using phosphoric acid or its derivatives as phosphorylation reagent is the degradation of cellulosic substrates due to excessive acidity of the reaction medium. It has been shown that phosphate esters (PE) also act as phosphorylating agents without causing significant substrate deterioration. Phosporylation reaction with PE has been performed on bleached Kraft fibers (KF) and lignocellulosic fibers from manufacturing of thermomechanical pulp. Moreover, similar results can be obtained using recycled lignocellulosic fibers or any cellulosic substrates from agricultural or forestry activity.

[004] It has been shown that cellulosic substrate can be phosphorylated with phosphate esters (PE) in the presence of an excess of urea following the reaction principle, as proposed by Inagaki *et al.* (1976) and Pieschel *et al.* (2004). Theoretically, it is possible to graft one phosphate moiety for each of the three hydroxyls of anhydroglucose unit of cellulose, therefore providing a KF:PE molar

ratio of 1:3 in most cases. Urea, which fulfills multiple functions in phosphorylation, can be largely added in excess, the KF:urea molar ratio being 1:17. As described in Shi *et al.* (2014), the phosphorylation reaction is conducted up to three hours in an anhydrous medium, at an average temperature of 150 °C, which slightly exceeds the melting point of urea. Finally, the fibers are washed thoroughly with water and ethanol and air dried.

[005] There is thus a need to provide alternative processes or reactants for phosphorylation and/or to improve at least one aspect of the prior art processes.

SUMMARY OF THE DISCLOSURE

[006] According to an aspect of the present disclosure, there is provided a lignocellulosic material comprising phosphorylated lignocellulosic fibers, wherein the fibers have an ionic charge in water of about 4000 to about 7000 mmoles/kg.

[007] According to another aspect of the present disclosure, there is provided a lignocellulosic material comprising phosphorylated lignocellulosic fibers, wherein the fibers have an average fiber length of about 0.5 to about 5 mm.

[008] According to another aspect of the present disclosure, there is provided a lignocellulosic material comprising phosphorylated lignocellulosic fibers.

[009] According to another aspect of the present disclosure, there is provided a process for preparing a phosphorylated lignocellulosic material comprising phosphorylated lignocellulosic fibers, the process comprising:

> reacting lignocellulosic fibers of a lignocellulosic material with a phosphate ester in the presence of urea.

[0010] According to another aspect of the present disclosure, there is provided a process for preparing a phosphorylated lignocellulosic material comprising phosphorylated lignocellulosic fibers, the process comprising:

reacting lignocellulosic fibers of a lignocellulosic material with a phosphate ester in the presence of an activating agent.

[0011] According to a further aspect of the present disclosure, there is provided a composition comprising:

a phosphate ester; and

at least one of a defoamer and a viscosity reducer.

[0012] Another aspect herein described relates to a composition comprising:

a phosphate ester; and

at least one of a C1-C12 alcohol and an ester of a carboxylic acid.

[0013] According to another aspect, there is provided the use of a composition as defined in the present disclosure as a phosphorylation reagent.

[0014] According to another aspect, there is provided the use of a composition as defined in the present disclosure for phosphorylating a lignocellulosic material.

[0015] According to another aspect, there is provided the use of a composition as defined in the present disclosure in the manufacture of phosphorylated lignocellulosic fibers.

[0016] According to another aspect, there is provided a method of using a composition according to the present disclosure. The method comprises reacting the composition with a lignocellulosic material comprising lignocellulosic fibers so as to at least partially phosphorylate the lignocellulosic fibers.

[0017] According to another aspect, there is provided a method for insulating a building comprising inserting or injecting into at least a wall, floor or ceiling of the

building the lignocellulosic material comprising phosphorylated lignocellulosic fibers of the present disclosure.

[0018] According to another aspect, there is provided a method for extracting heavy metal ions from an aqueous composition, said method comprising:

contacting said aqueous composition with the lignocellulosic material comprising phosphorylated lignocellulosic fibers of any one of claims 1 to 32 so as to obtain a mixture; and

filtering said mixture so as to separate the lignocellulosic material comprising phosphorylated lignocellulosic fibers comprising heavy metal ions connected thereto from said aqueous composition and recovering said aqueous composition at least partially depleted in heavy metal ions.

[0019] According to another aspect, there is provided a method for insulating a building comprising inserting or injecting into at least a wall, floor or ceiling of said building the lignocellulosic material comprising phosphorylated lignocellulosic fibers of the present disclosure.

[0020] According to another aspect, there is provided a method for manufacturing a peat moss product comprising mixing together peat moss and the lignocellulosic material comprising phosphorylated lignocellulosic fibers of the present disclosure.

[0021] According to another aspect, there is provided a composition comprising:

phosphorylated lignocellulosic fibers of any one of claims 1 to 32; and

peat moss.

[0022] According to another aspect, there is provided a composition comprising:

phosphorylated lignocellulosic fibers of any one of claims 1 to 32;

a surfactant; and

peat moss.

[0023] It has been found that the compositions and processes of the present disclosure are effective for preparing phosphorylated lignocellulosic fibers without however substantially damaging the fibers. In fact, it has been observed that by using the compositions and/or processes of the present disclosure, it is possible to carry out phosphorylation of lignocellulosic fibers by maintaining the integrity of the fibers. For example, the average length of the fibers can be substantially maintained. For example, the diameter of the fibers can be substantially maintained. For example, the fine content of the fibers can be substantially maintained.

DETAILED DESCRIPTION OF THE DISCLOSURE

[0024] Non-limiting examples of the present disclosure are hereby provided.

[0025] The terms “suitable” and “appropriate” mean that the selection of the particular group or conditions would depend on the specific synthetic manipulation to be performed and the identity of the molecule but the selection would be well within the skill of a person trained in the art. All process steps described herein are to be conducted under conditions suitable to provide the product shown. A person skilled in the art would understand that all reaction conditions, including, for example, reaction solvent, reaction time, reaction temperature, reaction pressure, reactant ratio, can be varied to optimize the yield of the desired product and it is within their skill to do so.

[0026] In understanding the scope of the present disclosure, the term “comprising” and its derivatives, as used herein, are intended to be open ended terms that specify the presence of the stated features, elements, components, groups, integers, and/or steps, but do not exclude the presence of other unstated features, elements, components, groups, integers and/or steps. The foregoing also

applies to words having similar meanings such as the terms, “including”, “having” and their derivatives. Finally, terms of degree such as “substantially”, “about” and “approximately” as used herein mean a reasonable amount of deviation of the modified term such that the end result is not significantly changed. These terms of degree should be construed as including a deviation of ±10% of the modified term if this deviation would not negate the meaning of the word it modifies.

[0027] As used in this specification and the appended claims, the singular forms “a”, “an” and “the” include plural references unless the content clearly dictates otherwise. Thus for example, a composition containing “a compound” includes a mixture of two or more compounds. It should also be noted that the term “or” is generally employed in its sense including “and/or” unless the content clearly dictates otherwise.

[0028] The definitions and embodiments described in particular sections are intended to be applicable to other embodiments herein described for which they are suitable as would be understood by a person skilled in the art.

[0029] The recitation of numerical ranges by endpoints herein includes all numbers and fractions subsumed within that range (e.g. 1 to 5 includes 1, 1.5, 2, 2.75, 3, 3.90, 4, and 5). It is also to be understood that all numbers and fractions thereof are presumed to be modified by the term "about."

[0030] The expression “substantially maintained”, when referring to a value of the average length of the lignocellulosic fibers, the diameter of the lignocellulosic fibers or the fine content of the lignocellulosic fibers refers to a value that is modified by a maximum of 10 % (i.e. modified by 10 % or less). For example, it was found that by using the compositions and/or processes of the present disclosure, it was possible to carry out phosphorylation of lignocellulosic fibers by substantially maintaining at least one of the value of the average length of the lignocellulosic fibers, the value of the diameter of the lignocellulosic fibers or the value of the fine content of the

lignocellulosic fibers. For example, it was found that pursuant to such processes or use of such compositions, the value of the average length of the phosphorylated lignocellulosic fibers, the value of the diameter of the phosphorylated lignocellulosic fibers or the value of the fine content of the phosphorylated lignocellulosic fibers is at least 90 % or 95 % of the corresponding value of the untreated or unphosphorylated fibers.

[0031] For example, the fibers can have an ionic charge in water of about 4500 to about 6500 mmoles/kg.

[0032] For example, the fibers can have an ionic charge in water of about 5000 to about 6500 mmoles/kg.

[0033] For example, the fibers can have an ionic charge in water of about 5000 to about 6000 mmoles/kg.

[0034] For example, the fibers can have an ionic charge in water of about 4000, about 4250, about 4500, about 4750, about 5000, about 5250, about 5500, about 5750, about 6000, about 6250, about 6500, about 6750, or about 7000 mmoles/kg.

[0035] For example, the fibers can have an average fiber length of about 0.5 to about 5 mm.

[0036] For example, the fibers can have an average fiber length of about 0.5 to about 4 mm.

[0037] For example, the fibers can have an average fiber length of about 0.8 to about 3 mm.

[0038] For example, the fibers can have an average fiber length of about 1 to about 4 mm.

[0039] For example, the fibers can have an average fiber length of about 0.5, about 0.6, about 0.7, about 0.8, about 0.9, about 1.0, about 1.1, about 1.2, about 1.3, about 1.4, about 1.5, about 1.6, about 1.7, about 1.8, about 1.9, about 2.0, about 2.1, about 2.2, about 2.3, about 2.4, about 2.5, about 2.6, about 2.7, about 2.8, about 2.9, about 3.0, about 3.1, about 3.2, about 3.3, about 3.4, about 3.5, about 3.6, about 3.7, about 3.8, about 3.9, about 4.0, about 4.1, about 4.2, about 4.3, about 4.4, about 4.5, about 4.6, about 4.7, about 4.8, about 4.9 or about 5.0 mm.

[0040] For example, the material, when reacted with water, is effective for absorbing about 30 to about 60 times its own weight of water.

[0041] For example, the material, when reacted with water, is effective for absorbing about 30 to about 50 times its own weight of water.

[0042] For example, the material, when reacted with water, is effective for absorbing about 35 to about 45 times its own weight of water.

[0043] For example, the material, when reacted with water, is effective for absorbing about 30, about 32, about 34, about 36, about 38, about 40, about 42, about 44, about 46, about 48 or about 50 times its own weight of water.

[0044] For example, the material has a phosphorus content of about 9 to about 17 % (w/w).

[0045] For example, the material has a phosphorus content of about 9 to about 15 % (w/w).

[0046] For example, the material has a phosphorus content of about 9 to about 13 % (w/w).

[0047] For example, the material has a phosphorus content of about 9, about 10, about 11, about 12, about 13, about 14 or about 15 % (w/w).

[0048] For example, the material has a Limit Oxygen Index of at least about 23.

[0049] For example, the material has a Limit Oxygen Index of at least about 25.

[0050] For example, the material has a Limit Oxygen Index of at least about 27.

[0051] For example, the material has a Limit Oxygen Index of at least about 25, at least about 27, at least about 30, at least about 35, at least about 40, at least about 45, at least about 50, at least about 55 or at least about 60.

[0052] For example, the material has a Limit Oxygen Index of about 25 to about 75.

[0053] For example, the material has a Limit Oxygen Index of about 25 to about 60.

[0054] For example, the material has a Limit Oxygen Index of about 27 to about 60.

[0055] For example, the material has a Limit Oxygen Index of about 30 to about 60.

[0056] For example, the material has a Limit Oxygen Index of about 25 to about 65.

[0057] For example, the material has a Limit Oxygen Index of about 25 to about 60.

[0058] For example, the material has a Limit Oxygen Index of about 25 to about 55.

[0059] For example, the material has a metal adsorption capacity of at least 1.7 mmoles/g for at least one heavy metal.

[0060] For example, the material has a metal adsorption capacity of about 1.7 to about 2.9 mmoles/g for at least one heavy metal.

[0061] For example, the material has a metal adsorption capacity of about 1.5 to about 3.5 mmoles/g for at least one heavy metal.

[0062] For example, the material has a metal adsorption capacity of about 1.5, about 1.6, about 1.7, about 1.8, about 1.9, about 2.0, about 2.1, about 2.2, about 2.3, about 2.4, about 2.5, about 2.6, about 2.7, about 2.8, about 2.9, about 3.0, about 3.1, about 3.2, about 3.3, about 3.4 or about 3.5 mmoles/g for at least one heavy metal.

[0063] For example, the at least one heavy metal is chosen from Ni, Cu, Cd and Pb.

[0064] For example, the at least one heavy metal is chosen from Fe, Cu, Mn, Co, Ni and Cd.

[0065] For example, the lignocellulosic material is effective for extracting at least 90 % of at least one heavy metal contained in a waste water that has a concentration of about 1 to about 1000 ppm in said at least one heavy metal.

[0066] For example, the lignocellulosic material is effective for extracting at least 95 % of at least one heavy metal contained in a waste water that has a concentration of about 1 to about 1000 ppm in said at least one heavy metal.

[0067] For example, the lignocellulosic material is effective for extracting about 100 % of at least one heavy metal contained in a waste water that has a concentration of about 1 to about 1000 ppm in said at least one heavy metal.

[0068] For example, the fibers are phosphorylated Kraft fibers.

[0069] For example, the fibers can be hybrid phosphorylated Kraft fibers comprising an ammonium ion and/or a sodium ion and hydrogen counter-ions on phosphate groups (for example on substantially each phosphate group).

[0070] For example, the lignocellulosic material has a charge excess of 10% based on the total ionic charge of the metal ions present in the material.

[0071] For example, the fibers can be acid phosphorylated Kraft fibers having two hydrogen counter-ions on phosphate groups (for example on substantially each phosphate group).

[0072] For example, the fibers can be sodium phosphorylated Kraft fibers having two sodium counter-ions on the phosphate groups (for example on substantially each phosphate group).

[0073] The person skilled in the art will readily understand that urea can be used as an *in situ* ammonia generation agent. Other suitable agents that generate ammonia can be used as well.

[0074] For example, the lignocellulosic material is a sheet, a panel, wood or fibers.

[0075] For example, the process can comprise reacting the lignocellulosic fibers of a lignocellulosic material with a mixture comprising the phosphate ester and an activation agent.

[0076] For example, the process can comprise reacting the lignocellulosic fibers of a lignocellulosic material with a mixture comprising the phosphate ester and urea.

[0077] For example, the mixture has a molar ratio, the phosphate ester / urea of about 1 / about 2 to about 6.

[0078] For example, the phosphate ester / urea molar ratio is about 1 / about 2, about 1 / about 2.5, about 1 / about 3, about 1 / about 3.5, about 1 / about 4, about 1 / about 4.5, about 1 / about 5, about 1 / about 5.5, or about 1 / about 6.

[0079] For example, the process can comprise reacting the lignocellulosic fibers of a lignocellulosic material with a mixture comprising the phosphate ester and urea at a molar ratio lignocellulosic fibers / phosphate ester / urea of about 1 / about 1.1 to about 4 / about 5 to about 20.

[0080] For example, the lignocellulosic fibers / phosphate ester / urea molar ratio can be about about 1 / about 1.1 / about 5, about 1 / about 1.1 / about 6, about 1 / about 1.1 / about 7, about 1 / about 1.1 / about 8, about 1 / about 1.1 / about 9, about 1 / about 1.1 / about 10, about 1 / about 1.1 / about 12, about 1 / about 1.1 / about 14, about 1 / about 1.1 / about 16, about 1 / about 1.1 / about 18, about 1 / about 1.1 / about 20, 1 / about 2 / about 5, about 1 / about 2 / about 5, about 1 / about 3 / about 5, about 1 / about 4 / about 5, about 1 / about 2 / about 6, about 1 / about 2 / about 7, about 1 / about 2 / about 8, about 1 / about 2 / about 10, about 1 / about 2 / about 12, about 1 / about 2 / about 14, about 1 / about 2 / about 16, about 1 / about 2 / about 18, about 1 / about 2 / about 20, 1 / about 4 / about 5, about 1 / about 4 / about 5, about 1 / about 3 / about 5, about 1 / about 4 / about 5, about 1 / about 4 / about 6, about 1 / about 4 / about 7, about 1 / about 4 / about 8, about 1 / about 4 / about 10, about 1 / about 4 / about 12, about 1 / about 4 / about 14, about 1 / about 4 / about 16, about 1 / about 4 / about 18, or about 1 / about 4 / about 20,

[0081] For example, the mixture can be at a temperature of about 125 to about 185 °C.

[0082] For example, the mixture can be at a temperature of about 130 to about 180 °C.

[0083] For example, the mixture can be at a temperature of about 140 to about 170 °C.

[0084] For example, the mixture can be at a temperature of about 125, about 130, about 135, about 140, about 145, about 150, about 155, about 160, about 165, about 170, about 175, about 180, about 185 or about 190 °C.

[0085] For example, the mixture further comprises at least one of a defoamer and a viscosity reducer.

[0086] For example, the mixture further comprises at least one C1-C12 alcohol.

[0087] For example, the mixture further comprises at least one C1-C6 alcohol.

[0088] For example, the mixture further comprises at least one ester of a carboxylic acid.

[0089] For example, the mixture further comprises at least one ester of a C1-C22 carboxylic acid.

[0090] For example, the mixture further comprises at least one ester of a C1-C16 carboxylic acid.

[0091] For example, the mixture further comprises at least one ester of a C1-C12 carboxylic acid.

[0092] For example, the mixture further comprises at least one ester of a C6-C12 carboxylic acid.

[0093] For example, the mixture further comprises at least one ester of a C8-C12 carboxylic acid.

[0094] For example, the at least one ester is a methyl, ethyl, propyl, butyl, pentyl or hexyl ester of the carboxylic acid.

[0095] For example, the at least one ester is a C1-C12 ester of the carboxylic acid.

[0096] For example, the phosphate ester comprises at least 65, at least 66, at least 67, at least 68, at least 69, at least 70, at least 71, at least 72, at least 73, at least 74, at least 75, at least 76, at least 77, at least 78, at least 79 or at least 80 % (w/w) of the phosphate in the form of a mono-ester phosphate.

[0097] For example, the fibers are added into the mixture.

[0098] For example, the lignocellulosic material is a sheet, a panel, wood or fibers.

[0099] For example, the lignocellulosic material is a sheet, a panel or wood and the fibers are impregnated with the mixture.

[00100] For example, the mixture further comprises water.

[00101] For example, the phosphate ester is obtained by reacting phosphoric acid with a C1-C22 alcohol.

[00102] For example, the phosphate ester is obtained by reacting phosphoric acid with a C1-C12 alcohol.

[00103] For example, the phosphate ester is a phosphate ester chosen from C6-C22 phosphate esters.

[00104] For example, the phosphate ester is a phosphate ester chosen from C6-C18 phosphate esters.

[00105] For example, the phosphate ester is a C12 phosphate ester.

[00106] For example, the phosphorylated fibers are hydrogen ammonium phosphate.

[00107] For example, the phosphorylated fibers are phosphorylated Kraft fibers.

[00108] For example, the process further comprises treating phosphorylated fibers with HCl.

[00109] For example, the process further comprises treating phosphorylated fibers with NaOH or KOH.

[00110] For example, the process provides a phosphorus content of at least 9, at least 10, at least 11, at least 12, at least 13, at least 14 or at least 15 % (w/w) to the phosphorylated lignocellulosic material.

[00111] For example, the process provides a phosphorus content of about 9 to about 15 % (w/w) to the phosphorylated lignocellulosic material.

[00112] For example, the process provides a phosphorus content of about 9 to about 13 % (w/w) to the phosphorylated lignocellulosic material.

[00113] The process herein described provides an increase in the phosphorylation yield. The skilled person will readily understand that the phosphorylation yield represents the weight gain of the fiber substrate after phosphorylation.

[00114] For example, the process provides a phosphorylation yield of at least 15, at least 20, at least 25, at least 30, at least 35, at least 40, at least 45 or at least 50 %.

[00115] For example, the process provides a phosphorylation yield of about 20 to about 50 %.

[00116] For example, the process provides a phosphorylation yield of about 22 to about 45 %.

[00117] The presently described process further provides an increase in the phosphorylation efficiency which readily understood as the amount of phosphorus from phosphate esters that is grafted to the fibers.

[00118] For example, the process provides a phosphorylation efficiency of at least 20, at least 21, at least 22, at least 23, at least 24, at least 25, at least 26, at least 27, at least 28, at least 29, at least 30, at least 31, at least 32, at least 33, at least 34, at least 35, at least 36, at least 37, at least 38, at least 39, at least 40, at least 41, at least 42, at least 43, at least 44, at least 45, at least 46, at least 47, at least 48, at least 49, at least 50, at least 51, at least 52, at least 53, at least 54, at least 55, at least 56, at least 57, at least 58, at least 59, at least 60, at least 61, at least 62, at least 63, at least 64, at least 65 or at least 70 %.

[00119] For example, the process provides a phosphorylation efficiency of about 20 to about 70 %.

[00120] For example, the process provides a phosphorylation efficiency of about 20 to about 60 %.

[00121] For example, the process provides a phosphorylation efficiency of about 20 to about 60 %.

[00122] For example, the process provides a phosphorylation efficiency of about 30 to about 60 %.

[00123] For example, the process provides a phosphorylation efficiency of about 29 to about 56 %.

[00124] For example, the process provides a phosphorylation efficiency of about 43 to about 56 %.

[00125] The lignocellulosic materials comprising phosphorylated lignocellulosic fibers as described herein can be used in various applications.

[00126] For example, the lignocellulosic material comprising phosphorylated lignocellulosic fibers is used in the manufacture of a fireproof material.

[00127] For example, the lignocellulosic material comprising phosphorylated lignocellulosic fibers is used as a fireproof material.

[00128] For example, the lignocellulosic material comprising phosphorylated lignocellulosic fibers is used for trapping at least one metal.

[00129] For example, the lignocellulosic material comprising phosphorylated lignocellulosic fibers is used for carrying out ion exchange.

[00130] For example, the lignocellulosic material comprising phosphorylated lignocellulosic fibers is used for water absorption.

[00131] For example, the lignocellulosic material comprising phosphorylated lignocellulosic fibers is used as a hydrogel.

[00132] For example, the lignocellulosic material comprising phosphorylated lignocellulosic fibers is used for waste water treatment.

[00133] For example, the lignocellulosic material comprising phosphorylated lignocellulosic fibers is used for papermaking.

[00134] For example, the lignocellulosic material comprising phosphorylated lignocellulosic fibers is used in diaper manufacturing.

[00135] For example, the lignocellulosic material comprising phosphorylated lignocellulosic fibers is used in the manufacture of a wood-based panel or fiber-based panel.

[00136] For example, the wood-based panel is chosen from high density fiberboards, medium density fiberboards, particle board, laminated wood, plywood and Wood Plastic Composite (WPC).

[00137] For example, the lignocellulosic material comprising phosphorylated lignocellulosic fibers is used in the manufacture of peat moss.

[00138] For example, the lignocellulosic material comprising phosphorylated lignocellulosic fibers is used in the manufacture of a peat moss product or a peat moss composition.

[00139] For example, the lignocellulosic material comprising phosphorylated lignocellulosic fibers is used together with a surfactant in the manufacture of a peat moss product or a peat moss composition.

[00140] For example, the lignocellulosic material comprising phosphorylated lignocellulosic fibers is used in the manufacture of a peat moss product or a peat moss composition.

[00141] For example, the lignocellulosic material comprising phosphorylated lignocellulosic fibers is used as a wetting agent in the manufacture of a peat moss product.

[00142] For example, the lignocellulosic material comprising phosphorylated lignocellulosic fibers is used for wetting peat moss.

[00143] For example, the lignocellulosic material comprising phosphorylated lignocellulosic fibers is used as a wetting agent.

[00144] For example, the lignocellulosic material comprising phosphorylated lignocellulosic fibers is usedin the manufacture of a heat insulation material.

[00145] For example, the lignocellulosic material comprising phosphorylated lignocellulosic fibers is used in the manufacture of a sound insulation material.

[00146] For example, the lignocellulosic material comprising phosphorylated lignocellulosic fibers is used in the manufacture of an anti-adhesive surface.

[00147] The present disclosure further provides reagents that can be used for the phosphorylation reaction of lignocellulosic fibers of lignocellulosic materials.

[00148] For example, the phosphate ester is a phosphate ester chosen from C6-C22 phosphate esters.

[00149] For example, the phosphate ester is a phosphate ester chosen from C6-C18 phosphate esters.

[00150] For example, the phosphate ester is a C12 phosphate ester.

[00151] For example, the composition comprises less than 20, less than 19, less than 18, less than 17, less than 16, less than 15, less than 14, less than 13, less than 12, less than 11, less than 10, less than 9, less than 8, less than 7, less than 6 or less than 5 % (w/w) of the phosphate ester in the form of a di-ester.

[00152] For example, the composition comprises at least 70, at least 71, at least 72, at least 73, at least 74, at least 75, at least 76, at least 77, at least 78, at least 79, at least 80, at least 81, at least 82, at least 83, at least 84, at least 85, at least 86, at least 87, at least 88, at least 89 or at least 90 % (w/w) of the phosphate ester in the form of a mono-ester.

[00153] For example, the composition comprises less than 10, less than 9, less than 8, less than 7, less than 6 or less than 5 % (w/w) of phosphoric acid.

[00154] For example, the composition comprises about 0.1 to about 15 %, about 1 to about 15 %, about 1 to about 12 %, about 1 to about 10 %, or about 2 to about 10 % (w/w) of a C1-C6 alcohol.

[00155] For example, the composition comprises about 0.1, about 0.2, about 0.3, about 0.4, about 0.5, about 0.6, about 0.7, about 0.8, about 0.9, about 1, about 2, about 3, about 4, about 5, about 6, about 7, about 8, about 9, about 10, about 11, about 12, about 13, about 14 or about 15 % (w/w) of a C1-C6 alcohol.

[00156] For example, the C1-C6 alcohol is methanol or ethanol.

[00157] For example, the composition comprises about 0.1 to about 15 %, about 1 to about 15 %, about 1 to about 12 %, about 1 to about 10 %, or about 2 to about 10 % (w/w) of an ester of a carboxylic acid.

[00158] For example, the composition comprises about 0.1, about 0.2, about 0.3, about 0.4, about 0.5, about 0.6, about 0.7, about 0.8, about 0.9, about 1, about 2, about 3, about 4, about 5, about 6, about 7, about 8, about 9, about 10, about 11, about 12, about 13, about 14 or about 15 % (w/w) of an ester of a carboxylic acid.

[00159] For example, the carboxylic acid is a C1-C22 carboxylic acid.

[00160] For example, the carboxylic acid is a C1-C16 carboxylic acid.

[00161] For example, the carboxylic acid is a C6-C12 carboxylic acid.

[00162] For example, the carboxylic acid is a C8-C12 carboxylic acid.

[00163] For example, the at least one ester of a carboxylic acid is a methyl, ethyl, propyl, butyl, pentyl or hexyl ester of the carboxylic acid.

[00164] For example, the at least one ester of a carboxylic acid is a C1-C12 ester of the carboxylic acid.

[00165] For example, the composition is used as a phosphorylation reagent.

[00166] For example, the composition is used for phosphorylating a lignocellulosic material.

[00167] For example, the composition is used in the manufacture of phosphorylated lignocellulosic fibers.

[00168] For example, wherein the method of use of the composition comprise diluting the composition with water and then impregnating said lignocellulosic with said diluted composition.

[00169] For example, the diluted composition comprises about 25 to about 45 % w/w of said composition.

[00170] For example, the diluted composition comprises about 30 to about 40 % w/w of said composition.

[00171] For example, in method for extracting heavy metal ions from an aqueous composition, the mixture has a pH of about 1 to about 4, about 2 to about 4 or about 2 to about 3.

[00172] For example, in method for extracting heavy metal ions from an aqueous composition, the mixture has a pH of about 1 to about 4, about 2 to about 4 or about 2 to about 3.

[00173] For example, the composition comprises about 70 to about 95 %, about 75 to about 95 %, about 85 to about 95 % or about 75 to about 90 % by weight of peat moss.

[00174] For example, the composition comprises about 5 to about 30 %, about 5 to about 20 %, about 5 to about 15 % or about 10 to about 25 % by weight of the lignocellulosic material comprising phosphorylated lignocellulosic fibers,

[00175] For example, the composition comprises a surfactant. For example, the surfactant can have dosage of at a dosage of about 200 to about 1200 mL/m^3, about 400 to about 1000 mL/m^3 or about 600 to about 800 mL/m^3

[00176] It will be appreciated by a person skilled in the art that embodiments relating to the lignocellulosic materials and the processes of preparation and uses thereof, as well compositions herein described can be varied as detailed herein.

[00177] The following examples are non-limitative and are used to better exemplify the materials and processes of the present disclosure.

EXAMPLES

Product characterization

[00178] It was found that the compositions and processes of the present disclosure are effective for preparing phosphorylated lignocellulosic fibers without however substantially damaging the fibers. In fact, it was observed that by using the compositions and processes of the present disclosure, it was possible to carry out phosphorylation of lignocellulosic fibers by maintaining the integrity of the fibers. For example, it was found that the average length of the fibers was substantially maintained. For example, it was found that the diameter of the fibers was substantially maintained. For example, it was found that the fine content of the fibers was substantially maintained.

[00179] Preliminary attempts of phosphorylation reaction have been made using two linear and saturated phosphate esters, namely with 8 carbons (PEC8) and 18 carbons (PEC18) in the aliphatic chain. Such teachings of phosphate esters synthesis, purification and characterization; phosphorylation pathway; phosphorylated Kraft fibers (KFP) characterization and properties; are largely described in Shi *et al.* (2014) and Shi *et al.* (2015). These papers also describe analyses which can be used to easily assess the efficiency of PEs as new phosphorylation reagents, including the following:

[00180] *Phosphorylation yield* [η (%)] which represents the weight gain of the cellulose substrate after phosphorylation:

$$\eta = \frac{w_{KFP} - w_{KF}}{w_{KF}} \times 100$$

$$where \begin{cases} w_{KF} : weight\ of\ Kraft\ fibers\ before\ phosphorylation\ (g) \\ w_{KFP} : weight\ of\ Kraft\ fibers\ after\ phosphorylation\ (g) \end{cases}$$

[00181] *Phosphorus content* [P (%)] which is calculated in two steps: the KFP sample is firstly digested and then the phosphate concentration is measured by ultraviolet-visible (UV-Vis) absorption spectroscopy. A complete description of this method is described in Belosinschi (2014).

$$P = 0.0653 \times \frac{c_{PO_4^{2-}}}{w_{KFP}}$$

$$\text{where} \begin{cases} c_{PO_4^{2-}} & : \textit{phosphate concentration}\,(mg/L) \\ w_{KFP} & : \textit{weight of KFP sample}\,(g) \end{cases}$$

[00182] *Phosphorylation efficiency* [E (%)] which shows how much of phosphorus from PE is finally grafted to the KF substrate:

$$E = \frac{DS}{R_{PE/KF}} \times 100$$

$$\text{where} \begin{cases} DS & : \textit{degree of substitution of KFP sample} \\ R_{PE/KF} & : \textit{molar ratio of phosphate ester to Kraft fibers} \end{cases}$$

[00183] The substitution degree (DS) which is related to the phosphorus content (P) of KFP sample by the following relation:

$$DS = \frac{162 \times P}{3100 - 97 \times P}$$

Materials and methods

Raw materials

[00184] The phosphorylation reaction can be carried out on any substrate containing a significant amount of cellulose. The substrates which were evaluated are wood (pine and maple), wood pulp fibers (chemical, thermomechanical and recycled) and plant fibers (cotton and flax).

[00185] The cellulosic substrate can be treated in various forms including dust, pulps, sheets, panels or wood. As such, the application technique of the reagent will vary depending on the form of the substrate.

Reagents

[00186] In addition to the cellulosic substrate, the phosphorylation reaction involves the following reagents:

1. Phosphate ester: Linear and saturated phosphate esters in their acid form, with the aliphatic chain length between 6 and 18 carbon atoms. Preferably, the phosphate ester has a composition of more than 70% (w/w) mono-ester phosphate, less than 20% (w/w) di-ester phosphate and a maximum of 10% (w/w) unreacted phosphoric acid.
2. Urea: Is added in excess with regard to phosphate esters. The added amount (molar ratio) of urea is 2 to 6 times more than the phosphate ester used.
3. Short chain alcohol (methanol or ethanol): These chemical compounds act as solvents, defoamers and viscosity reducers and up to 15% (w/w) were introduced in the phosphate ester formulations.
4. Carboxylic esters: These chemical compounds act as defoamers and viscosity reducers and up to 20% (w/w) were introduced in the phosphate ester formulations. The carboxylic esters with an aliphatic chain length between 8 and 12 carbon atoms, e.g. methyl octanoate (C1C8 carboxylic acid ester) or methyl dodecanoate (C1C12 carboxylic acid ester), can be mixed to a synthesized phosphate ester.

Reaction with fibers

[00187] The phosphate ester and urea are heated in an oven at a temperature of about 140 ^{o}C to about 170 ^{o}C. The fibers are added to the phosphate ester/urea blend under constant mixing to obtain a homogenous contact of all reagents. The reaction is conducted for a minimum of one hour and a maximum of three hours in

this anhydrous environment. Finally, the fibers are washed thoroughly with water and ethanol and air dried.

Reaction with a sheet, a panel or wood

[00188] The same composition of phosphorylation reagent is applicable whether the reaction is performed on sheets, panels, wood or fibers. In order to preserve the pre-set structure of sheets, panels and wood, the substrate impregnation with phosphorylation reagent is done by filtration at room temperature. However, the urea is previously dissolved in water since it is solid at room temperature. Therefore, an impregnation solution is made by mixing about 30 to about 40% (w/w) phosphorylation reagent or composition with water. The reaction period increases in this case because the evaporation of water takes extra time.

Post-reaction treatments

[00189] After the reaction, the fibers are obtained in a hybrid form (hydrogen ammonium phosphate). At this point, the fibers can be used for flame retardant and ion exchange applications. For other applications such as water absorption or ion exchange, the performance of the product can be improved by changing the counter-ions of the grafted phosphate. Subsequent treatments with diluted aqueous solution of acid (HCl) and alkali (NaOH) allow to obtain the phosphorylated fibers in their sodium form. Scheme 1 below represents chemical reactions previously discussed during preparation of phosphorylated kraft fibers and their different forms.

Scheme 1. Synthesis of KFP and different forms thereof

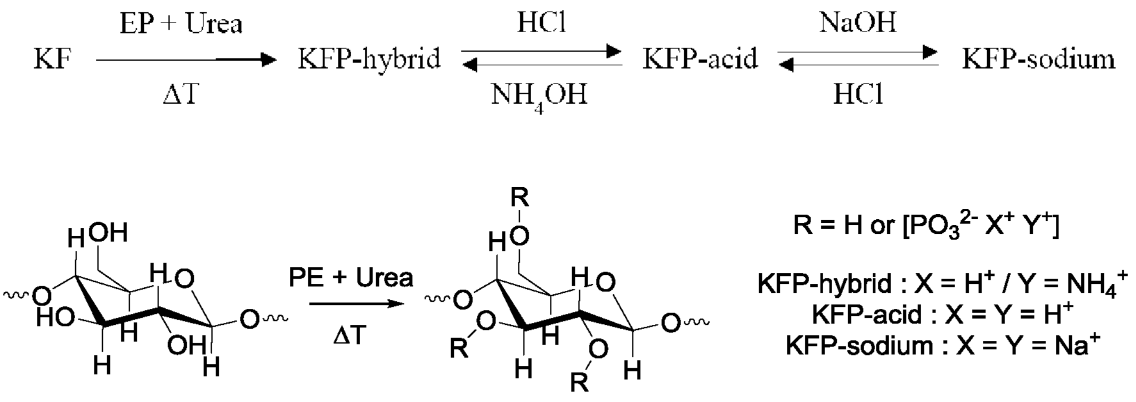

Results and discussion

[00190] Table 1 shows the characteristics of phosphorylated Kraft fiber (KFP) samples obtained using PEC8 and PEC18 as phosphorylation reagents. These results clearly confirm the potential of phosphate esters to react with cellulose substrates. For example, the KFP with a phosphorus content up to 9% can be adequate for some applications like flame retardant. However, only 23% of phosphorylation efficiency can eventually cause environmental issues. Therefore, new recipes based on phosphate esters were tested in order to maximize the phosphorylation results.

Table 1. Phosphorylation results using phosphate esters

	PEC8	PEC18
η (%)	19	11
P (%)	9.23	7.17
E (%)	22.61	16.11

[00191] As can be seen in Table 1, the phosphorylation yield (η (%)), phosphorus content (P (%)) and phosphorylation efficiency (E (%)) appear to decrease upon increasing the aliphatic chain length of phosphate ester. Without wishing to be bound by such a theory, it was found that this trend is mainly due to the viscosity of

phosphate esters which increases as the aliphatic chain length increases. The capacity of the Kraft fibers to soak a more viscous phosphate ester appears to decrease with phosphorylation with PEC18.

[00192] In order to reduce reagent viscosity, the phosphate esters were ethoxylated. An average of three ethoxylate molecules were bound to two phosphate esters, with 10 and 12 carbons in the aliphatic chain. The phosphorylation results with these reagents, namely PEC10C6 and PEC12C6 respectively, are shown in Table 2.

Table 2. Phosphorylation results using ethoxylated phosphate esters

	PEC10C6	PEC12C6
η (%)	29	22
P (%)	10.86	9.73
E (%)	28.65	24.37

[00193] The ethoxylation of phosphate esters improves the phosphorylation process when comparing Table 2 vs. Table 1 results. Yet, the ethoxyl moiety does not interfere in the phosphorylation mechanism, it only reduces the viscosity of the reaction medium. It appears that a low viscosity of the phosphate ester reagents is a prerequisite for improving the overall efficiency of phosphorylation reaction.

[00194] In addition, chemical compounds acting as defoamers and viscosity reducers were introduced in the phosphate ester formulations. Methanol (CH_3OH), up to 15% (w/w), and methyl dodecanoate (C1C12 carboxylic acid ester), up to 20% (w/w), were mixed to a synthesized phosphate ester with 12 carbons in the aliphatic chain (PEC12). This time, the PEC12 was obtained by reacting a fatty alcohol 1-dodecanol (C12OH) with polyphosphoric acid (115% H_3PO_4 basis). The choice of polyphosphoric acid over phosphorus pentoxide was made so as to avoid the formation of diester phosphate as by-product (Tracy *et al.*, 2002) which further increases the viscosity of synthesized PEC12.

[00195] Table 3 shows the phosphorylation results of Kraft fibers with the PEC12 mixture at different molar ratios. The PEC12 mixture is the first phosphorylation reagent with a phosphorylation efficiency exceeding 50%. Accordingly, the water pollution is significantly reduced as the amount of phosphate discharged during the KFP washing stage is limited. Another major advantage is that the phosphorylation results can be easily modulated by changing the molar ratio of reactants. The amount of phosphate ester used can be chosen depending on the final application of phosphorylated fibers. This limits the wastes and allows a more judicious use of reagents.

Table 3. Phosphorylation results using viscosity reducers/phosphate ester mixture

KF / PEC12 / urea (molar ratio)	1 / 1.5 / 8.5	1 / 2 / 11.5	1 / 3 / 17
η (%)	22	36	45
P (%)	9.73	11.79	12.91
E (%)	56.06	56.15	43.41

[00196] These changes in phosphate ester formulation simplify the phosphorylation process and ultimately increase the reaction yield and efficiency. Finally, almost all the chemical compounds contained in the PEC12 mixture, i.e. methanol, methyl dodecanoate and 1-dodecanol can be biosourced.

Various applications

[00197] ***Flame retardant.*** The phosphorylated lignocellulosic fibers can act themselves as a flame retardant or can be part of manufacturing of fireproof materials. The LOI (Limit Oxygen Index) values of phosphorylated Kraft fibers with two phosphate esters are shown in Table 4. As can be seen, all LOI values are greater than 27, regardless the form of phosphorylated fibers and the phosphorylation reagent. A material is considered an excellent flame retardant if the

LOI is greater than 25. The LOI value for untreated cellulosic fibers is 19, which means that it burns easily.

Table 4. LOI values for the phosphorylated Kraft fibers with PEC8 and PEC18

**Fiber form**	PEC8	PEC18
Hydrogen ammonium	57	30
Dihydrogen	35	27
Disodium	29	28

[00198] ***Ion exchange.*** The phosphorylated Kraft fibers are characterized by a high anionic charge in water (up to 5000-6000 mmoles/Kg) which indicates advantageous adsorption capacity of heavy metal cations. Table 5 shows the adsorption capacity of phosphorylated Kraft fibers for different cations. The results of two commercially available ion exchange resins (Dowex™ 50WX2-400 and Dowex™ Marathon C) are also presented for comparison.

Table 5. Adsorption capacity (mmoles/g) of KFP forms for different heavy metals

	KFP-hybrid	KFP-acid	KFP-sodium	Dowex™ 50WX2-400	Dowex™ Marathon C
Ni	2.4	1.9	2.8	2.4	2.5
Cu	2.5	2.0	2.8	2.4	2.5
Cd	2.3	1.7	2.7	2.5	2.5
Pb	2.7	2.5	2.9	2.5	2.6

[00199] The adsorption capacities of phosphorylated Kraft fibers are comparable to those of commercially available resins. Actually, the cation adsorption capacity is slightly lower for the KFP-acid form, comparable for the -hybrid form and higher for the -sodium form than that of the two evaluated commercially available ion exchange resins.

[00200] ***Wastewater treatment.*** KFP have been used to extract heavy metal ions from an industrial process water. The sample, obtained from the mining industry, contained heavy metal ions and hydrofluoric acid (pH 2.4). The total amount of metal ions found in the sample was determined by ICP-OES. An amount of KFP, hybrid form, corresponding to a charge excess of 10% (based on the total ionic charge of the metal ions present in the sample) was added to the wastewater sample. It was stirred at low speed for 30 minutes at 20°C. The fibers were then filtered and the filtrate was analyzed by ICP-OES to determine the removal efficiency for several metal ions. The results are shown in Table 6. All tested heavy metals were removed from the solution (93-100% removal) except for chromium which was in anionic form (negatively charged) at pH 2.4.

Table 6. Removal efficiency of KFP hybrid form for different heavy metals found in a mining industry process water

Metal ions	Cu	Fe	Mn	Co	Ni	Cr	Cd
Concentration before treatment (ppm)	22	830	10.5	3.6	226	77	0
Concentration after treatment (ppm)	< 0.5	1	< 0.5	< 0.5	16	45	0
Removal efficiency (%)	100	100	100	100	93	42	100

[00201] ***Water absorption.*** One of the main properties of phosphorylated Kraft fibers is the great polarity generated by grafting phosphate moieties. These groups have an absorption capacity of water much higher than the equivalent hydroxyl groups of cellulose. In some conditions, the phosphate groups may be at the origin of the formation of a hydrogel based phosphorylated fibers. The results obtained so far show that this hydrogel is capable to retain up to 40 times more water than its weight. Possible applications for these highly water absorbent fibers include for example papermaking (mainly paper towels), diaper manufacturing, as well as crop controlled irrigation in dry areas.

[00202] ***Peat moss wetting.*** KFP have been used as a wetting and water retention agent in a peat moss sample. The sample was obtained from a commercial

distributor. Two different surfactants (similar to those used in the peat moss industry) were added to the sample at a dosage of 800 mL/m^3. Table 7 reports an improvement of about 50% in water absorption with these surfactants. Another sample was mixed with KFP (90% peat moss / 10% KFP). In this case, the amount of water retained in the sample was almost doubled (94% improvement) and the water was retained more strongly in the peat moss (higher resistance to drainage by pressure). Finally, to evaluate a possible synergistic effect between the surfactants and KFP, two more samples of the 90% peat moss / 10% KFP were treated with 800 mL/m^3 of each surfactant. In this case, the additional gain in water absorption obtained with the surfactants is about 20 to about 30%.

Table 7. Water absorption capacity of a commercial peat moss sample treated with KFP and different surfactants

Sample	Water absorption (g water / g peat moss)
Peat Moss alone	1,97
Peat Moss + Surfactant 1	3,01
Peat Moss + Surfactant 2	2,89
Peat Moss + KFP	3,82
Peat Moss + KFP + Surfactant 1	4,29
Peat Moss + KFP + Surfactant 2	4,01

[00203] *Heat & Sound Insulation*. Due to their high anionic charge, KFP have a tendency to repulse each other thus forming a very open structure with a low specific volume. They also have a very high elastic return after compression. These properties make KFP a potential insulation material. It is well known that lignocellulosic fibers have a very low thermal conductivity (about 0.045 W/mK for cellulose). The thermal conductivity of KFP is very similar to untreated fibers. However, because of the very low bulk of KPF mats, the actual amount of fibers needed to obtain the same mat thickness (and same thermal conductivity) as untreated fibers is much lower. Kraft (KF) and KFP mats (22 cm x 22 cm) were produced with the same mass of fibers. The thermal conductivity of the two mats is reported in TABLE 8. The thermal

conductivity (W/mK) is similar for KF and KFP. However, the KFP mat is 25% thicker. In consequence, a 25% reduction of the amount of fiber is possible with KFP for the same insulating effect.

TABLE 8. Thermal Conductivity Tests Made on KF and KFP

Sample	Thickness (mm)	Thermal Conductivity (W/mK)
KF	2,62	0,034
KFP	3,28	0,032

[00204] The embodiments of paragraphs [0023] to [00203] of the present disclosure are presented in such a manner in the present disclosure so as to demonstrate that every combination of embodiments, when applicable, can be made. These embodiments have thus been presented in the description in a manner equivalent to making dependent claims for all the embodiments that depend upon any of the preceding claims (covering the previously presented embodiments), thereby demonstrating that they can be combined together in all possible manners. For example, all the possible combinations, when applicable, between the various embodiments of paragraphs [0023] to [00203] as well as the various embodiments of paragraphs [006] to [0022] are hereby covered by the present disclosure.

REFERENCES

Inagaki, N., Nakamura, S., Asai, H., Katsuura, K. Phosphorylation of cellulose with phosphorous acid and thermal degradation of the product. Journal of Applied Polymer Science, 20 (10), 2829-2836, (1976).

Pieschel, F., Lange, E., Camacho, J., Körber, H. Starch phosphates method for the production thereof and their use. United States patent, (2004).

Shi, Y., Belosinschi, D., Brouillette, F., Belfkira, A., Chabot, B. Phosphorylation of Kraft fibers with phosphate esters. Carbohydrate Polymers, 106, 121-127, (2014).

Shi, Y., Belosinschi, D., Brouillette, F., Belfkira, A., Chabot, B. The properties of phosphorylated Kraft Fibers. BioResources, 10 (3), 4375-4390, (2015).

Belosinschi, D. Coating of phosphate esters dispersions for the production of release paper. Doctoral thesis (in French). Quebec University at Trois-Rivieres, (2014).

Tracy, J.D., Reierson, L.R. Commercial synthesis of monoalkyl phosphates. Journal of surfactants and detergents, 5 (2), 169-172, (2002).

WHAT IS CLAIMED IS:

1. A lignocellulosic material comprising phosphorylated lignocellulosic fibers, wherein said fibers have an ionic charge in water of about 4000 to about 7000 mmoles/kg.

2. The lignocellulosic material of claim 1, wherein said fibers have an ionic charge in water of about 4500 to about 6500 mmoles/kg.

3. The lignocellulosic material of claim 1, wherein said fibers have an ionic charge in water of about 5000 to about 6500 mmoles/kg

4. The lignocellulosic material of claim 1, wherein said fibers have an ionic charge in water of about 5000 to about 6000 mmoles/kg.

5. The lignocellulosic material of any one of claims 1 to 4, wherein said fibers have an average fiber length of about 0.5 to about 5 mm.

6. A lignocellulosic material comprising phosphorylated lignocellulosic fibers, wherein said fibers have an average fiber length of about 0.5 to about 5 mm.

7. The lignocellulosic material of any one of claims 1 to 6, wherein said fibers have an average fiber length of about 0.8 to about 3 mm.

8. The lignocellulosic material of any one of claims 1 to 7, wherein said material, when reacted with water, is effective for absorbing about 30 to about 50 times its own weight of water.

9. The lignocellulosic material of any one of claims 1 to 7, wherein said material, when reacted with water, is effective for absorbing about 35 to about 45 times its own weight of water.

10. The lignocellulosic material of any one of claims 1 to 9, wherein said material has a phosphorus content of about 9 to about 15 % (w/w).

11. The lignocellulosic material of any one of claims 1 to 9, wherein said material has a phosphorus content of about 9 to about 13 % (w/w).

12. The lignocellulosic material of any one of claims 1 to 11, wherein said material has a Limit Oxygen Index value of at least about 27.

13. The lignocellulosic material of any one of claims 1 to 11, wherein said material has a Limit Oxygen Index value of about 27 to about 60.

14. The lignocellulosic material of any one of claims 1 to 11, wherein said material has a Limit Oxygen Index value of about 25 to about 75.

15. The lignocellulosic material of any one of claims 1 to 14, wherein said lignocellulosic material has a Limit Oxygen Index value of about 25 to about 65.

16. The lignocellulosic material of any one of claims 1 to 14, wherein said lignocellulosic material has a Limit Oxygen Index value of about 25 to about 60.

17. The lignocellulosic material of any one of claims 1 to 14, wherein said lignocellulosic material has a Limit Oxygen Index value of about 25 to about 55.

18. The lignocellulosic material of any one of claims 1 to 17, wherein said material has a metal adsorption capacity of at least 1.7 mmoles/g for at least one heavy metal.

19. The lignocellulosic material of any one of claims 1 to 18, wherein said material has a metal adsorption capacity of about 1.7 to about 2.9 mmoles/g for at least one heavy metal.

20. The lignocellulosic material of any one of claims 1 to 18, wherein said material has a metal adsorption capacity of about 1.5 to about 3.5 mmoles/g for at least one heavy metal.

21. The lignocellulosic material of any one of claims 18 to 20, wherein said at least one heavy metal is chosen from Ni, Cu, Cd and Pb.

22. The lignocellulosic material of any one of claims 18 to 20, wherein said at least one heavy metal is chosen from Fe, Cu, Mn, Co, Ni and Cd.

23. The lignocellulosic material of any one of claims 1 to 17, wherein said material is effective for extracting at least 90 % of at least one heavy metal contained in a waste water that has a concentration of about 1 to about 1000 ppm in said at least one heavy metal.

24. The lignocellulosic material of any one of claims 1 to 17, wherein said material is effective for extracting at least 95 % of at least one heavy metal contained in a waste water that has a concentration of about 1 to about 1000 ppm in said at least one heavy metal.

25. The lignocellulosic material of any one of claims 1 to 17, wherein said material is effective for extracting about 100 % of at least one heavy metal contained in a waste water that has a concentration of about 1 to about 1000 ppm in said at least one heavy metal.

26. The lignocellulosic material of any one of claims claim 23 to 25, wherein said at least one heavy metal is chosen from Fe, Cu, Mn, Co, Ni and Cd.

27. The lignocellulosic material of any one of claims 1 to 26, wherein said fibers are phosphorylated Kraft fibers.

28. The lignocellulosic material of any one of claims 1 to 27, wherein said fibers are hybrid phosphorylated Kraft fibers having an ammonium ion and/or a sodium ion and hydrogen counter-ions on substantially each phosphate group.

29. The lignocellulosic material of claim 28, wherein said material has a charge excess of 10% based on the total ionic charge of the metal ions present in the material.

30. The lignocellulosic material of any one of claims 1 to 27, wherein said fibers are acid phosphorylated Kraft fibers having two hydrogen counter-ions on substantially each phosphate group.

31. The lignocellulosic material of any one of claims 1 to 27, wherein said fibers are sodium phosphorylated Kraft fibers having two sodium counter-ions on substantially each phosphate group.

32. The lignocellulosic material of any one of claims 1 to 27, wherein said lignocellulosic material is a sheet, a panel, wood or fibers.

33. A process for preparing a phosphorylated lignocellulosic material comprising phosphorylated lignocellulosic fibers, said process comprising:

reacting lignocellulosic fibers of a lignocellulosic material with a phosphate ester in the presence of urea.

34. The process of claim 33, wherein said process comprises reacting said lignocellulosic fibers of a lignocellulosic material with a mixture comprising said phosphate ester and urea.

35. The process of claim 34, wherein said mixture has a molar ratio, said phosphate ester / urea of about 1 / about 2 to about 6.

36. The process of claim 34, wherein said process comprises reacting said lignocellulosic fibers of a lignocellulosic material with a mixture comprising said phosphate ester and urea at a molar ratio lignocellulosic fibers / phosphate ester / urea of about 1 / about 1.1 to about 4 / about 5 to about 20.

37. The process of claim 34, 35 or 36, wherein said mixture is at a temperature of about 125 to about 185 °C.

38. The process of claim 34, 35 or 36, wherein said mixture is at a temperature of about 130 to about 180 °C.

39. The process of claim 34, 35 or 36, wherein said mixture is at a temperature of about 140 to about 170 °C.

40. The process of any one of claims 34 to 39, wherein said mixture further comprises at least one of a defoamer and a viscosity reducer.

41. The process of any one of claims 34 to 39, wherein said mixture further comprises at least one C1-C12 alcohol.

42. The process of any one of claims 34 to 39, wherein said mixture further comprises at least one C1-C6 alcohol.

43. The process of any one of claims 34 to 42, wherein said mixture further comprises at least one ester of a carboxylic acid.

44. The process of any one of claims 34 to 42, wherein said mixture further comprises at least one ester of a C1-C22 carboxylic acid.

45. The process of any one of claims 34 to 42, wherein said mixture further comprises at least one ester of a C1-C16 carboxylic acid.

46. The process of any one of claims 34 to 42, wherein said mixture further comprises at least one ester of a C1-C12 carboxylic acid.

47. The process of any one of claims 34 to 42, wherein said mixture further comprises at least one ester of a C6-C12 carboxylic acid.

48. The process of any one of claims 34 to 42, wherein said mixture further comprises at least one ester of a C8-C12 carboxylic acid.

49. The process of any one of claims 46 to 48, wherein said at least one ester is a methyl, ethyl, propyl, butyl, pentyl or hexyl ester of said carboxylic acid.

50. The process of any one of claims 46 to 48, wherein said at least one ester is a C1-C12 ester of said carboxylic acid.

51. The process of any one of claims 34 to 50, wherein said phosphate ester comprises at least 65 % of said phosphate in the form of a mono-ester phosphate.

52. The process of any one of claims 34 to 50, wherein said phosphate ester comprises at least 70 % of said phosphate in the form of a mono-ester phosphate.

53. The process of any one of claims 34 to 50, wherein said phosphate ester comprises at least 75 % of said phosphate in the form of a mono-ester phosphate.

54. The process of any one of claims 34 to 53, wherein said fibers are added into said mixture.

55. The process of any one of claims 34 to 53, wherein said lignocellulosic material is a sheet, a panel, wood or fibers.

56. The process of any one of claims 34 to 53, wherein said lignocellulosic material is a sheet, a panel or wood and the fibers are impregnated with said mixture.

57. The process of claim 56, wherein said mixture further comprises water.

58. The process of any one of claims 33 to 57, wherein said phosphate ester is obtained by reacting phosphoric acid with a C1-C22 alcohol.

59. The process of any one of claims 33 to 57, wherein said phosphate ester is obtained by reacting phosphoric acid with a C1-C12 alcohol.

60. The process of any one of claims 33 to 59, wherein said phosphate ester is a phosphate ester chosen from C6-C22 phosphate esters.

61. The process of any one of claims 33 to 59 wherein said phosphate ester is a phosphate ester chosen from C6-C18 phosphate esters.

62. The process of any one of claims 33 to 59, wherein said phosphate ester is a C12 phosphate ester.

63. The process of any one of claims 33 to 59, wherein said phosphorylated fibers are hydrogen ammonium phosphate.

64. The process of any one of claims 33 to 63, wherein said phosphorylated fibers are phosphorylated Kraft fibers.

65. The process of any one of claims 33 to 64, further comprising treating said phosphorylated fibers with HCl.

66. The process of any one of claims 33 to 65, further comprising treating said phosphorylated fibers with NaOH or KOH.

67. The process of any one of claims 33 to 65, wherein said process provides a phosphorus content of at least 9% (w/w) to said phosphorylated lignocellulosic material.

68. The process of any one of claims 33 to 65, wherein said process provides a phosphorus content of about 9 to about 15 % (w/w) to said phosphorylated lignocellulosic material.

69. The process of any one of claims 33 to 65, wherein said process provides a phosphorus content of about 9 to about 13 % (w/w) to said phosphorylated lignocellulosic material.

70. The process of any one of claims 33 to 69, wherein said process provides a phosphorylation yield of at least 15%.

71. The process of any one of claims 33 to 69, wherein said process provides a phosphorylation yield of about 20 to about 50 %.

72. The process of any one of claims 33 to 69, wherein said process provides a phosphorylation yield of about 22 to about 45 %.

73. The process of any one of claims 33 to 72, wherein said process provides a phosphorylation efficiency of at least 20%.

74. The process of any one of claims 33 to 72, wherein said process provides a phosphorylation efficiency of about 20 to about 70 %.

75. The process of any one of claims 33 to 72, wherein said process provides a phosphorylation efficiency of about 20 to about 60 %.

76. The process of any one of claims 33 to 72, wherein said process provides a phosphorylation efficiency of about 20 to about 60 %.

77. The process of any one of claims 33 to 72, wherein said process provides a phosphorylation efficiency of about 30 to about 60 %.

78. The process of any one of claims 33 to 72, wherein said process provides a phosphorylation efficiency of about 29 to about 56 %.

79. The process of any one of claims 33 to 72, wherein said process provides a phosphorylation efficiency of about 43 to about 56 %.

80. Use of the lignocellulosic material comprising phosphorylated lignocellulosic fibers of any one of claims 1 to 32 in the manufacture of a fireproof material.

81. Use of the lignocellulosic material comprising phosphorylated lignocellulosic fibers of any one of claims 1 to 32 as a fireproof material.

82. Use of the lignocellulosic material comprising phosphorylated lignocellulosic fibers of any one of claims 1 to 32 as for trapping at least one metal.

83. Use of the lignocellulosic material comprising phosphorylated lignocellulosic fibers of any one of claims 1 to 32 as carrying out ion exchange.

84. Use of the lignocellulosic material comprising phosphorylated lignocellulosic fibers of any one of claims 1 to 32 for water absorption.

85. Use of the lignocellulosic material comprising phosphorylated lignocellulosic fibers of any one of claims 1 to 32 as a hydrogel.

86. Use of the lignocellulosic material comprising phosphorylated lignocellulosic fibers of any one of claims 1 to 32 for waste water treatment.

87. Use of the lignocellulosic material comprising phosphorylated lignocellulosic fibers of any one of claims 1 to 32 for papermaking.

88. Use of the lignocellulosic material comprising phosphorylated lignocellulosic fibers of any one of claims 1 to 32 in diaper manufacturing.

89. Use of the lignocellulosic material comprising phosphorylated lignocellulosic fibers of any one of claims 1 to 32 in the manufacture of a wood-based panel or fiber-based panel.

90. The use of claim 89, wherein said wood-based panel is chosen from high density fiberboards, medium density fiberboards, particle board, laminated wood, plywood and Wood Plastic Composite (WPC).

91. Use of the lignocellulosic material comprising phosphorylated lignocellulosic fibers of any one of claims 1 to 32 in the manufacture of peat moss.

92. Use of the lignocellulosic material comprising phosphorylated lignocellulosic fibers of any one of claims 1 to 32 in the manufacture of a peat moss product or a peat moss composition.

93. Use of the lignocellulosic material comprising phosphorylated lignocellulosic fibers of any one of claims 1 to 32 and a surfactant in the manufacture of a peat moss product or a peat moss composition.

94. Use of the lignocellulosic material comprising phosphorylated lignocellulosic fibers of any one of claims 1 to 32 as a wetting agent in the manufacture of a peat moss product.

95. Use of the lignocellulosic material comprising phosphorylated lignocellulosic fibers of any one of claims 1 to 32 for wetting peat moss.

96. Use of the lignocellulosic material comprising phosphorylated lignocellulosic fibers of any one of claims 1 to 32 as a wetting agent.

97. Use of the lignocellulosic material comprising phosphorylated lignocellulosic fibers of any one of claims 1 to 32 in the manufacture of a heat insulation material.

98. Use of the lignocellulosic material comprising phosphorylated lignocellulosic fibers of any one of claims 1 to 32 in the manufacture of a sound insulation material.

99. Use of the lignocellulosic material comprising phosphorylated lignocellulosic fibers of any one of claims 1 to 32 in the manufacture of an anti-adhesive surface.

100. A method for insulating a building comprising inserting or injecting into at least a wall, floor or ceiling of said building the lignocellulosic material comprising phosphorylated lignocellulosic fibers of any one of claims 1 to 32.

101. A method for extracting heavy metal ions from an aqueous composition, said method comprising:

contacting said aqueous composition with the lignocellulosic material comprising phosphorylated lignocellulosic fibers of any one of claims 1 to 32 so as to obtain a mixture; and

filtering said mixture so as to separate the lignocellulosic material comprising phosphorylated lignocellulosic fibers comprising heavy metal ions connected thereto from said aqueous composition and recovering said aqueous composition at least partially depleted in heavy metal ions.

102. The method of claim 101, wherein said mixture has a pH of about 1 to about 4.

103. The method of claim 101, comprising maintaining pH at a value of about 2 to about 4.

104. The method of claim 101, comprising maintaining pH at a value of about 2 to about 3.

105. A method for insulating a building comprising inserting or injecting into at least a wall, floor or ceiling of said building the lignocellulosic material comprising phosphorylated lignocellulosic fibers of any one of claims 1 to 32.

106. A method for manufacturing a peat moss product comprising mixing together peat moss and the lignocellulosic material comprising phosphorylated lignocellulosic fibers of any one of claims 1 to 32.

107. The method of claim 106, comprising mixing together about 70 to about 95 % by weight of peat moss and about 5 to 30 % of the lignocellulosic material comprising phosphorylated lignocellulosic fibers of any one of claims 1 to 32.

108. The method of claim 106, further comprising adding a surfactant.

109. The method of claim 106, further comprising adding a surfactant at a dosage of at a dosage of about 200 to 1200 mL/m^3.

110. The method of claim 106, further comprising adding a surfactant at a dosage of at a dosage of about 600 to 800 mL/m^3.

111. A composition comprising:

phosphorylated lignocellulosic fibers of any one of claims 1 to 32; and

peat moss.

112. A composition comprising:

phosphorylated lignocellulosic fibers of any one of claims 1 to 32;

a surfactant; and

peat moss.

113. The composition of claim 111 or 112, wherein the composition comprises about 70 to about 95 % by weight of peat moss.

114. The composition of claim 111 or 112, wherein the composition comprises about 5 to about 30 % of the lignocellulosic material comprising phosphorylated lignocellulosic fibers of any one of claims 1 to 32.

115. A composition comprising:

a phosphate ester; and

at least one of a defoamer and a viscosity reducer.

116. A composition comprising:

a phosphate ester; and

at least one of a C1-C12 alcohol and an ester of a carboxylic acid.

117. The composition of claim 115 or 116, wherein said phosphate ester is a phosphate ester chosen from C6-C22 phosphate esters.

118. The composition of claim 115 or 116, wherein said phosphate ester is a phosphate ester chosen from C6-C18 phosphate esters.

119. The composition of claim 115 or 116, wherein said phosphate ester is a C12 phosphate ester.

120. The composition of any one of claims 115 to 119, wherein said composition comprises less than 20 % (w/w) of said phosphate ester in the form of a di-ester.

121. The composition of any one of claims 115 to 119, wherein said composition comprises less than 10 % (w/w) of said phosphate ester in the form of a di-ester.

122. The composition of any one of claims 115 to 121, wherein said composition comprises at least 70 % (w/w) of said phosphate ester in the form of a mono-ester.

123. The composition of any one of claims 115 to 121, wherein said composition comprises at least 80 % (w/w) of said phosphate ester in the form of a mono-ester.

124. The composition of any one of claims 115 to 123, wherein said composition comprises less than 10 % (w/w) of phosphoric acid.

125. The composition of any one of claims 115 to 123, wherein said composition comprises less than 5 % (w/w) of phosphoric acid.

126. The composition of any one of claims 115 to 125, wherein said composition comprises about 0.1 to about 15 % (w/w) of a C1-C6 alcohol.

127. The composition of any one of claims 115 to 125, wherein said composition comprises about 0.1 to about 15 % (w/w) of a methanol or ethanol.

128. The composition of any one of claims 115 to 127, wherein said composition comprises about 0.1 to about 15 % (w/w) of at least one ester of a carboxylic acid.

129. The composition of any one of claims 115 to 127, wherein said composition comprises about 0.1 to about 15 % (w/w) of an ester of a C1-C22 carboxylic acid.

130. The composition of any one of claims 115 to 127, wherein said composition comprises about 0.1 to about 15 % (w/w) of an ester of a C1-C16 carboxylic acid.

131. The composition of any one of claims 115 to 127, wherein said composition comprises about 0.1 to about 15 % (w/w) of an ester of a C1-C12 carboxylic acid.

132. The composition of any one of claims 115 to 127, wherein said composition comprises about 0.1 to about 15 % (w/w) of an ester of a C6-C12 carboxylic acid.

133. The composition of any one of claims 115 to 127, wherein said composition comprises about 0.1 to about 15 % (w/w) of an ester of a C8-C12 carboxylic acid.

134. The composition of any one of claims 128 to 133, wherein said ester of said carboxylic acid is a methyl, ethyl, propyl, butyl, pentyl or hexyl ester of said carboxylic acid.

135. The composition of any one of claims 128 to 133, wherein said ester of carboxylic said acid is a C1-C12 ester of said carboxylic acid.

136. Use of the composition of any one of claims 115 to 135, as a phosphorylation reagent.

137. Use of the composition of any one of claims 115 to 135 for phosphorylating a lignocellulosic material.

138. Use of the composition of any one of claims 115 to 135 in the manufacture of phosphorylated lignocellulosic fibers.

139. A method of using the composition of any one of claims 115 to 135, said method comprises reacting said composition with a lignocellulosic material comprising lignocellulosic fibers so as to at least partially phosphorylate said lignocellulosic fibers.

140. The method of claim 139, wherein said method comprises diluting said composition with water and then impregnating said lignocellulosic with said diluted composition.

141. The method of claim 139, wherein said diluted composition comprises about 25 to about 45 % w/w of said composition.

142. The method of claim 139, wherein said diluted composition comprises about 30 to about 40 % w/w of said composition.

INTERNATIONAL SEARCH REPORT

International application No.
PCT/CA2017/050717

A. CLASSIFICATION OF SUBJECT MATTER
IPC: ***C08H 8/00*** (2010.01) , ***A61L 15/28*** (2006.01) , ***B01J 20/22*** (2006.01) , ***C02F 1/28*** (2006.01)

According to International Patent Classification (IPC) or to both national classification and IPC

B. FIELDS SEARCHED

Minimum documentation searched (classification system followed by classification symbols)
C08H 8/00 (2010.01) , ***A61L 15/28*** (2006.01) , ***B01J 20/22*** (2006.01) , ***C02F 1/28*** (2006.01)

Documentation searched other than minimum documentation to the extent that such documents are included in the fields searched

Electronic database(s) consulted during the international search (name of database(s) and, where practicable, search terms used)
Canadian Patent Database, Questel Orbit, Scopus
Keywords: lignocellulose, phosphate, phosphoryl, phosphorylate, phosphorylation, kraft, peat moss, ionic charge, viscosity reducer, phosphate ester, defoamer, methanol, carboxylic acid

C. DOCUMENTS CONSIDERED TO BE RELEVANT

Category*	Citation of document, with indication, where appropriate, of the relevant passages	Relevant to claim No.
X	Shi et al., "The Properties of Phosphorylated Kraft Fibers"; *BioResources*, 2015, Vol. 10(3), pp.4375-4390<br>Abstract<br>Page 4376, lines 1-24<br>Page 4376, lines 25-35<br>Page 4376, lines 36-44<br>Page 4386<br>Table 2<br>Table 3	1-39, 51-87, 89-90, 96, 97 and 100-105

☑ Further documents are listed in the continuation of Box C. ☑ See patent family annex.

*	Special categories of cited documents:	"T"	later document published after the international filing date or priority date and not in conflict with the application but cited to understand the principle or theory underlying the invention
"A"	document defining the general state of the art which is not considered to be of particular relevance	"X"	document of particular relevance; the claimed invention cannot be considered novel or cannot be considered to involve an inventive step when the document is taken alone
"E"	earlier application or patent but published on or after the international filing date	"Y"	document of particular relevance; the claimed invention cannot be considered to involve an inventive step when the document is combined with one or more other such documents, such combination being obvious to a person skilled in the art
"L"	document which may throw doubts on priority claim(s) or which is cited to establish the publication date of another citation or other special reason (as specified)	"&"	document member of the same patent family
"O"	document referring to an oral disclosure, use, exhibition or other means		
"P"	document published prior to the international filing date but later than the priority date claimed		

Date of the actual completion of the international search<br>24 August 2017 (24-08-2017)	Date of mailing of the international search report<br>05 September 2017 (05-09-2017)
Name and mailing address of the ISA/CA<br>Canadian Intellectual Property Office<br>Place du Portage I, C114 - 1st Floor, Box PCT<br>50 Victoria Street<br>Gatineau, Quebec K1A 0C9<br>Facsimile No.: 819-953-2476	Authorized officer<br>Pierre Tessier (819) 639-9392

Box No. II Observations where certain claims were found unsearchable (Continuation of item 2 of the first sheet)

This international search report has not been established in respect of certain claims under Article 17(2)(a) for the following reasons:

1. ☐ Claim Nos.:
because they relate to subject matter not required to be searched by this Authority, namely:

2. ☐ Claim Nos.:
because they relate to parts of the international application that do not comply with the prescribed requirements to such an extent that no meaningful international search can be carried out, specifically:

3. ☐ Claim Nos.:
because they are dependent claims and are not drafted in accordance with the second and third sentences of Rule 6.4(a).

Box No. III Observations where unity of invention is lacking (Continuation of item 3 of first sheet)

This International Searching Authority found multiple inventions in this international application, as follows:

Group A – Claims 1-114 are directed to phosphorylated lignocellulosic fibers with a ionic charge in water of 4000-7000 or an average fiber length of 0.5-5 mm, processes for preparing said fibers and use of said fibers in fireproofing, insulation, for trapping metals, for water absorption, as hydrogels, for waste water treatment, for papermaking and for diaper, peat moss, anti-adhesive, sound or heat insulation or wood-panel manufacture;

Group B – Claims 115 (full) and 117-142 (in part) are directed to compositions comprising a phosphate ester and a defoamer or viscosity reducer and use of said composition as a phosphorylation reagent;

Group C – Claims 116 (full) and 117-142 (in part) are directed to compositions comprising a phosphate ester and a C1-C12 alcohol or an ester of a carboxylic acid and use of said composition as a phosphorylation reagent.

1. ☐ As all required additional search fees were timely paid by the applicant, this international search report covers all searchable claims.

2. ☑ As all searchable claims could be searched without effort justifying additional fees, this Authority did not invite payment of additional fees.

3. ☐ As only some of the required additional search fees were timely paid by the applicant, this international search report covers only those claims for which fees were paid, specifically claim Nos.:

4. ☐ No required additional search fees were timely paid by the applicant. Consequently, this international search report is restricted to the invention first mentioned in the claims; it is covered by claim Nos.: 1-114

Remark on Protest

☐ The additional search fees were accompanied by the applicant's protest and, where applicable, the payment of a protest fee.

☐ The additional search fees were accompanied by the applicant's protest but the applicable protest fee was not paid within the time limit specified in the invitation.

☐ No protest accompanied the payment of additional search fees.

C (Continuation). DOCUMENTS CONSIDERED TO BE RELEVANT

Category*	Citation of document, with indication, where appropriate, of the relevant passages	Relevant to claim No.
X	Shi et al., "Phosphorylation of Kraft Fibers with Phosphate Esters"; *Carbohydrate Polymers*, Vol. 106, pp.121-127<br>Abstract<br>Table 1<br>Section 3.1	1-39, 51-79, 116-128 and 136-142
X	CA 2 769 986 A1 (Chakrabarti et al.) 06 January 2011 (06-01-2011)<br>Examples<br>Page 12	115-127
X	CA 1 163 163 (Lappi et al.) 06 March 1984 (06-03-1984)<br>Abstract<br>Page 11, lines 10-26	115 and 117-125
X	US 5,882,541 (Actmann) 16 March 1999 (16-03-1999)<br>Column 4, line 62<br>Column 8, lines 39-42	115 and 123-125
X	CA 1 254 731 (Jones) 30 May 1989 (30-05-1989)<br>Abstract<br>Page 3, line 20<br>Page 10, lines 2-4	116-127
X	CA 1,335,578 (Fujio et al.) 16 May 1995 (16-05-1995)<br>Abstract<br>Page 3, lines 5-10	116-126 and 129-136
A	US 2013/0317138 A1 (Harada et al.) 28 November 2013 (28-11-2013)<br>Whole Document	1-114
A	US 3,459,588 (Davis) 05 August 1969 (05-08-1969)<br>Whole Document	1-114

INTERNATIONAL SEARCH REPORT
Information on patent family members

International application No.
PCT/CA2017/050717

Patent Document Cited in Search Report	Publication Date	Patent Family Member(s)	Publication Date
US3459588A	05 August 1969 (05-08-1969)	None	
US2013317138A1	28 November 2013 (28-11-2013)	US2013317138A1	28 November 2013 (28-11-2013)
		US8796363B2	05 August 2014 (05-08-2014)
		CN103415573A	27 November 2013 (27-11-2013)
		CN103415573B	27 April 2016 (27-04-2016)
		EP2681281A1	08 January 2014 (08-01-2014)
		EP2681281A4	14 January 2015 (14-01-2015)
		JP2012193337A	11 October 2012 (11-10-2012)
		JP5842526B2	13 January 2016 (13-01-2016)
		KR20130130850A	02 December 2013 (02-12-2013)
		KR101552414B1	10 September 2015 (10-09-2015)
		WO2012118165A1	07 September 2012 (07-09-2012)
CA2769986A1	06 January 2011 (06-01-2011)	CA2769986A1	06 January 2011 (06-01-2011)
		CN102482517A	30 May 2012 (30-05-2012)
		CN102482517B	29 June 2016 (29-06-2016)
		EP2449038A1	09 May 2012 (09-05-2012)
		JP2012532204A	13 December 2012 (13-12-2012)
		RU2012103931A	20 August 2013 (20-08-2013)
		US2012095128A1	19 April 2012 (19-04-2012)
		US9040624B2	26 May 2015 (26-05-2015)
		WO2011002474A1	06 January 2011 (06-01-2011)
CA1163163A	06 March 1984 (06-03-1984)	CA1163163A	06 March 1984 (06-03-1984)
		BR8108664A	25 May 1982 (25-05-1982)
		FI820599L	23 February 1982 (23-02-1982)
		FI74625B	30 November 1987 (30-11-1987)
		FI74625C	10 March 1988 (10-03-1988)
		GB2094287A	15 September 1982 (15-09-1982)
		GB2094287B	22 August 1984 (22-08-1984)
		NO820545A	22 February 1982 (22-02-1982)
		SE8201023L	18 February 1982 (18-02-1982)
		SE451382B	05 October 1987 (05-10-1987)
		US4445971A	01 May 1984 (01-05-1984)
		WO8200039A1	07 January 1982 (07-01-1982)
US5882541A	16 March 1999 (16-03-1999)	US5882541A	16 March 1999 (16-03-1999)
		AT291946T	15 April 2005 (15-04-2005)
		CA2270537A1	14 May 1998 (14-05-1998)
		CA2270537C	20 June 2006 (20-06-2006)
		DE69732915D1	04 May 2005 (04-05-2005)
		DE69732915T2	13 April 2006 (13-04-2006)
		EP0936938A1	25 August 1999 (25-08-1999)
		EP0936938A4	03 November 1999 (03-11-1999)
		EP0936938B1	30 March 2005 (30-03-2005)
		WO9819743A1	14 May 1998 (14-05-1998)
CA1254731A	30 May 1989 (30-05-1989)	None	
CA1335578C	16 May 1995 (16-05-1995)	CA1335578C	16 May 1995 (16-05-1995)
		DE3888307D1	14 April 1994 (14-04-1994)
		DE3888307T2	16 June 1994 (16-06-1994)
		EP0319130A2	07 June 1989 (07-06-1989)
		EP0319130A3	16 May 1990 (16-05-1990)
		EP0319130B1	09 March 1994 (09-03-1994)
		JPH01199590A	10 August 1989 (10-08-1989)
		JP2695180B2	24 December 1997 (24-12-1997)
		KR920009525B1	17 October 1992 (17-10-1992)
		US5212079A	18 May 1993 (18-05-1993)
